# Integration of pan-omics technologies and three-dimensional in vitro tumor models: an approach toward drug discovery and precision medicine

**DOI:** 10.1186/s12943-023-01916-6

**Published:** 2024-03-09

**Authors:** Anmi Jose, Pallavi Kulkarni, Jaya Thilakan, Murali Munisamy, Anvita Gupta Malhotra, Jitendra Singh, Ashok Kumar, Vivek M. Rangnekar, Neha Arya, Mahadev Rao

**Affiliations:** 1 Department of Pharmacy Practice, Manipal College of Pharmaceutical Sciences, Manipal Academy of Higher Education, Manipal, Karnataka 576104 India; 2 Department of Biochemistry, All India Institute of Medical Sciences Bhopal, Bhopal, Madhya Pradesh 462020 India; 3 Department of Translational Medicine, All India Institute of Medical Sciences Bhopal, Bhopal, Madhya Pradesh 462020 India; 4 Markey Cancer Center and Department of Radiation Medicine, University of Kentucky, Lexington, KY 40536 USA

**Keywords:** Multi-omics, Genomics, Transcriptomics, Lipidomics, Patient-derived organoids, 3-D in vitro tumor models, Precision medicine

## Abstract

Despite advancements in treatment protocols, cancer is one of the leading cause of deaths worldwide. Therefore, there is a need to identify newer and personalized therapeutic targets along with screening technologies to combat cancer. With the advent of pan-omics technologies, such as genomics, transcriptomics, proteomics, metabolomics, and lipidomics, the scientific community has witnessed an improved molecular and metabolomic understanding of various diseases, including cancer. In addition, three-dimensional (3-D) disease models have been efficiently utilized for understanding disease pathophysiology and as screening tools in drug discovery. An integrated approach utilizing pan-omics technologies and 3-D in vitro tumor models has led to improved understanding of the intricate network encompassing various signalling pathways and molecular cross-talk in solid tumors. In the present review, we underscore the current trends in omics technologies and highlight their role in understanding genotypic-phenotypic co-relation in cancer with respect to 3-D in vitro tumor models. We further discuss the challenges associated with omics technologies and provide our outlook on the future applications of these technologies in drug discovery and precision medicine for improved management of cancer.

**Graphical Abstract:**

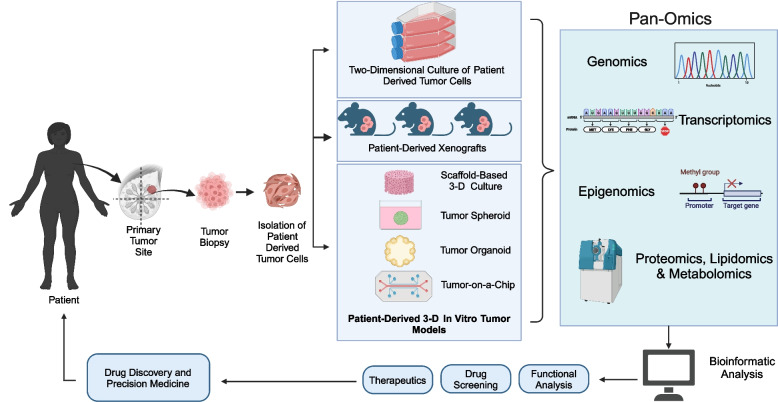

**Supplementary Information:**

The online version contains supplementary material available at 10.1186/s12943-023-01916-6.

## Introduction

Cancer is a global public health concern and accounts for approximately 10 million deaths worldwide [1]. According to the World Health Organization, estimated number of new cases will rise to 30.2 million by 2040 [1], thereby demanding newer drug targets, screening modalities for improved high-throughput drug discovery pipelines, and better treatment regimens for personalized medicine. In this regard, recent studies have utilized pan-omics technologies such as genomics, epigenomics, transcriptomics, proteomics, metabolomics, and lipidomics toward precise unravelling of disease pathophysiology as well as identifying newer drug targets. In addition, drug discovery and development encompass several stages that are associated with high costs as well as long duration [2]. Toward this, the recent decade has witnessed numerous high-throughput screening platforms for novel drug identification. Traditionally, drug screening and evaluation of anti-cancer drugs include investigations using conventional methods such as two-dimensional (2-D) culture of cancer cells and in vivo xenograft mouse models. The 2-D culture of cancer cells has the advantage of being simple and cost-effective and hence it is implemented in various functional tests for understanding disease pathophysiology and identification of new therapeutic targets [3]. However, these models do not mimic the native state of the tissue or the tumor since they lack appropriate cell–cell and cell-extracellular matrix (ECM) interaction [4]. Although, in vivo models demonstrate appropriate cell–cell and cell-ECM interaction they are linked with ethical constraints, high investments, increased incubation periods, and difficulty in discerning stages of developing metastasis. Therefore, there has been a paradigm shift toward the use of technologies that support the three-dimensional (3-D) culture of cancer cells under in vitro conditions. Tumor models based on the 3-D culture of cancer cells have been shown to recapitulate the complexities of the in vivo tumors such as hypoxia and cell–cell or cell-ECM interactions, toward improved genotype–phenotype relationship analyses [5–7]. In addition, cancer therapy has transitioned to individualized precision medicine approaches through identification of therapies based on the unique biology of patients and is dependent upon the prediction of unique molecular signatures that eventually drive optimal treatment regimen. Previous studies have demonstrated the integration of robust preclinical 3-D in vitro disease models with patient’s omics profiling toward identification of effective treatment strategies [8–10].

In the light of drug discovery and precision medicine, this review will discuss the potential application of integrating 3-D in vitro tumor models with pan-omics technologies for improved cancer therapy. The review will further elucidate various bioinformatic databases that can assist in the effective implementation of personalized therapies in cancer. Toward the end, an outlook demonstrating the application of pan-omics and 3-D in vitro tumor models relevant to drug development and precision medicine is highlighted.

## Omics and 3-D in vitro tumor models: focus on drug discovery

Increased costs of chemotherapeutic agents are attributed to the associated complexities of clinical trials and regulatory requirements. Moreover, unlike other drugs, chemotherapeutic drugs are associated with low success rates from bench side to the clinic often due to lack of translation of appropriate pre-clinical models. Drug discovery is a long process that encompasses a series of pre-clinical models and tests followed by clinical trials which demands physiologically relevant screening tools to improve the success rate. With the advent of multi-omics approach and 3-D in vitro tumor models, the time period between target discovery and clinical application of respective targeted therapy has significantly reduced [11].

In the search for new targets, evaluation of biological samples at the level of genes, transcripts, proteins, metabolites, and their interaction networks is now possible especially with the advent of omics technology [12]. In particular, genome-wide association studies (GWASs), whole genome sequencing, and transcriptome analysis represent crucial methods to identify and validate new pharmacological targets since they can offer a methodical approach to assess their therapeutic efficacy and associated adverse effects [13]. Recent developments in sequencing, microarray, and mass spectrometry (MS) technology enable researchers to understand the underlying molecular processes involved in complex diseases and determine therapeutic targets, understand their mode of action, and further evaluate the adverse effects. Eventually, the pan-omics technology and investigations offer crucial data for the delivery of tailored treatment.

Drug testing utilizing omics technology for 3-D culture models, such as 3-D spheroids, patient-derived explants, patient-derived organoids (PDO), scaffold-based cultures, 3-D bioprinted models and organ-on-a-chip models, offers a functional medicine perspective and is an essential component to genomic testing [14]. The ideal 3-D tumor model incorporates traits of the host's immune system and the tumor's genetic features in a tissue-specific context to replicate the tumor microenvironment. The tumor microenvironment is composed of the ECM and stromal cells including immune cells, cancer-associated fibroblasts, pericytes, endothelial cells, adipocytes, epithelial cells and nerve cells. Through the release of chemokines, growth factors, and regulatory molecules including microRNAs (miRNAs), the stromal cells interact with one another as well as the tumor cells, and contribute to tumor growth, proliferation, metastasis, and chemoresistance [15]. Therefore, it is critical to develop a 3-D tumor model based on the components of the tumor stroma for improved recapitulation of the parent tumor and for pre-clinical drug testing studies [16]. Integrating omics technologies with 3-D disease models to elucidate the complex links between genotype and phenotype of cancer cells have huge potential in terms of high throughput drug screening and drug discovery.

**Fig. 1:**
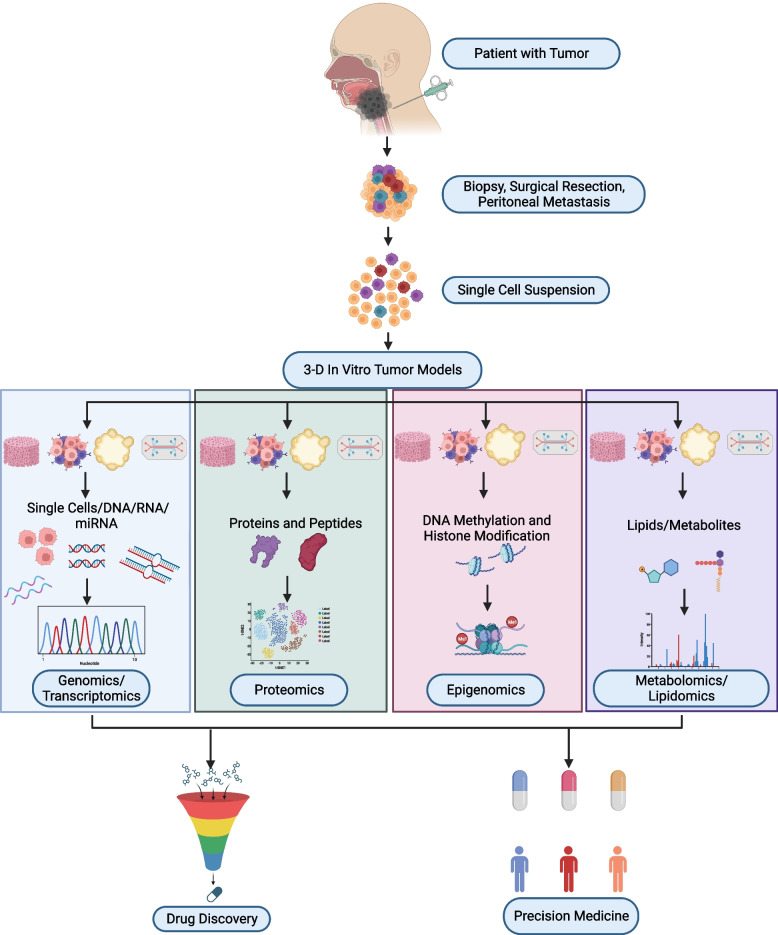
Integration of patient-derived 3-D in vitro tumor models and pan-omics techniques for drug discovery and precision medicine. The figure depicts the workflow for integration of pan-omics technology and 3-D in vitro tumor models for application in drug discovery and precision medicine. In the first step, 3-D in vitro tumor models are generated and can be based on scaffolds, 3-D spheroids, 3-D organoids and organ-on-a-chip models with the utilization of the tissue samples obtained from patient tissue biopsy, surgically resected tissues and peritoneal metastasis [17]. Thereafter, the 3-D in vitro tumor models are subjected to various omics techniques including genomics, transcriptomics, proteomics, lipidomics or metabolomics. The data generated by these pan-omics techniques is then filtered, aligned and analyzed using various bioinformatics tools and also compared with parent tumor tissue. The data can then be applied to discover novel therapeutic targets for diagnostic and therapeutic monitoring of cancer patients. The data can also be used to assist in clinical decision making for administration of chemotherapeutic agents thereby aiding in precision medicine. Figure created with BioRender.com

In the upcoming sections, we will discuss the role of various omics technologies in drug screening, drug discovery, and precision medicine using 3-D in vitro tumor models. Figure 1 depicts the amalgamation of 3-D in vitro tumor models and various omics approaches for drug discovery and precision medicine.

### Genomics and 3-D in vitro tumor models

Sequencing the target genome using various genomic methods such as DNA microarray, Sanger sequencing, next-generation sequencing (NGS), and third-generation of long reads sequencing (TGS) have been applied to elucidate the inter-individual changes at the somatic and germline level [18, 19]. With the advent of high-throughput sequencing methodologies, The Cancer Genome Atlas (TCGA) facilitated transformation and acceleration of personalized medicine by molecular characterization of over 20,000 primary cancers and matched normal samples across 33 cancer groups, and generated huge genomic, epigenomic, transcriptomic, and proteomic data, leading to the identification of potential therapeutic targets for cancer treatment [20]. Various researchers have reported the integration of genomics with 3-D in vitro tumor models in drug discovery and precision medicine through the identification of tumor biomarkers, gene expression profiling, single nucleotide polymorphism detection, genes associated with chemoresistance and prediction of drug response [21, 22]. Initial studies have integrated genomics data obtained using microarray technology for the characterization of 3-D models [23–28]. As an example, DNA microarray compared the gene expression profiles in a well-characterized breast cancer-based 3-D model with a 2-D monolayer culture following treatment with anti-cancer drugs such as doxorubicin, paclitaxel, and tamoxifen [23]. The 3-D model showed enhanced resistance to chemotherapy relative to the 2-D culture suggesting that the 3-D model recapitulated the cellular structure, phenotypic heterogeneity of cancer cells and the ECM barrier to drug transport, that eventually contributed to chemotherapy resistance [23]. Microarray has also been used to evaluate the role of hypoxia in 3-D tumor models and demonstrated prominent interdependence between culture dimensionality and hypoxia response, partially mediated by pro-inflammatory signalling pathways [24].

In addition, multiparametric genomic analysis using NGS has been utilized for disease subtype classification and comparison of the disease model to patient tumor data [29–34]. Several studies have used whole genome sequencing (WGS) and whole exome sequencing (WES) to identify the mutational landscape of tumor models and to confirm whether the model maintains molecular characteristics of the disease subtype [35–37]. The Englander Institute for Precision Medicine unveiled a platform technology using uterine and colon cancer patient-derived tumor organoid living biobank and integrated WES with high throughput drug screening to identify the most effective treatment options for individual patients [10]. In another study, Calandrini et al*.* reported organoid models of pediatric kidney cancer, which were characterized using histology, WGS, RNA sequencing (RNA-seq), DNA methylation profiling, and single-cell RNA-sequencing (scRNA-seq) analyses further supporting the integrative platform in patient-specific drug sensitivity for informed clinical decision making [38]. Recently, Cho et al. incorporated the application of genomics techniques and omics databases in a large set of colorectal cancer organoids (CCO) and identified the importance of intrinsic immuno-genomic characteristics that affect tumor immune microenvironment (TIME). The CCOs not only recapitulated the genetic profiles of primary tumors, but also identified two distinct intrinsic molecular subgroups of highly proliferative and mesenchymal phenotypes of colorectal cancer. Furthermore, authors discovered that TIME phenotype was associated with microsatellite instability, Wnt/β-catenin signalling, and APC/KRAS mutations thereby demonstrating the application of tumor organoids in developing novel immunological strategies for precise patient stratification [31]. In conclusion, living biobanks based on 3-D in vitro tumor models can provide novel therapeutic options not only for the subsequent assessment in clinical trials but also for adjuvant therapies that can further guide clinical decision-making for individual patients with limited clinical treatment options.

With the help of validated 3-D in vitro tumor models and sequencing approaches, it is now possible to identify the specific genomic drivers that contribute to drug sensitivity and therapeutic response [39–43]. One such study developed pancreatic cancer PDOs that mimicked the mutational spectrum, transcriptional subtypes of primary tumors as well as therapeutic response with patient outcomes thereby enabling prospective therapeutic selection [39]. In another study, Mitra et al*.* performed whole genome miRNA-microarray profiling in retinoblastoma cell line-based surface-engineered, biodegradable polymeric microparticles and revealed altered gene and miRNA expression such as upregulation of various oncogenes including MYCN, ERBB3, and IGFBP5 followed by overexpression of collagen, laminin and fibronectin; increased expression of ECM could cause variation in drug effects [40]. Moreover, an advanced organoid-based platform was established for pooled CRISPR-Cas9 screening, to carry out high-throughput genetic testing and functional identification of tumor drivers [41].

Another exciting application of integrating 3-D in vitro tumor models with NGS is to assist clinicians in their decision-making toward the identification of patients suitable for targeted therapies. Several NGS-based in vitro companion diagnostic tests (CDx) are currently used to identify eligible patients for PARPi therapies, considering BRCA1 and/or BRCA2 gene mutations. Patient-derived models are now frequently employed as a part of routine functional assays in clinical oncology to evaluate PARPi sensitivity and for the discovery of novel biomarkers to forecast patient clinical outcomes [44, 45]. A proposed integrative approach to evaluate PARPi sensitivity used dose–response testing on patient-derived tumor organoids (PDTO) in conjunction with NGS. In this regard, a perspective study proposed that X-ray exposure to PDTOs could be used to evaluate homologous recombination capacity and identify patients who could potentially be benefited from PARPi therapy [46].

In conclusion, sequencing tools along with NGS demonstrate potential in multiparametric genome analysis, identify genomic markers contributing to drug sensitivity, and aid clinicians in appropriate clinical decision-making for improved patient care.

### Transcriptomics and 3-D in vitro tumor models

Transcriptomics studies have primarily been driven by bulk RNA sequencing (RNA-seq) to validate various in vitro/in vivo models by comparing their profiles to the patient/parent tumor tissue [8, 47]. Since transcriptomics encompasses the post-transcriptional era, changes that are not discernible at the genomics level may become apparent at the transcriptomics level. Thus, in order to develop more dependable treatments, these alterations should be detected if they are pathogenically significant. Advanced techniques such as scRNA-seq provide a precise understanding of the genomic landscape in specific cancer patients for drug discovery and precision medicine [48].

Many studies have reported the transcriptomic analysis of PDOs to understand tumor heterogeneity, drug resistance and predict the response to chemotherapeutic drugs [49–55]. Transcriptomic studies enable a mechanistic understanding of tumor pathophysiology as well as treatment response [39, 54, 56, 57]. Mastri et al*.* studied the transcriptome-based molecular classifications in the bladder cancer patient-derived xenografts, organoids, and spheroids [58]. In another study, the authors explored ex vivo pharmacogenomic profiling of PDOs derived from liver metastasis of colorectal patients; herein, PDOs from 39 metastases from 22 individuals were further subjected to drug sensitivity testing with 40 therapeutically relevant drugs followed by transcriptomic analysis. The authors identified three drug-response clusters within the metastatic colorectal cancer groups; this was based on sensitivities to epidermal growth factor receptor (EGFR) and/or murine double minute 2 (MDM2) inhibition, and correlating with RAS mutations as well as TP53 activity. However, there was a limited intra-patient heterogeneity in drug sensitivity between multiple liver metastases PDOs [59]. Recently, a genotype–phenotype mapping study based on patient-derived lung organoids was used to understand the Wnt dependency in lung adenocarcinoma; authors demonstrated that the loss of the alveolar identity gene NKX2-1 increases the Wnt dependency in lung cancer, irrespective of the presence of EGFR mutation. The study highlighted the potential of lung cancer organoid screening for Wnt targeting therapy with EGFR screening and various therapeutic strategies to combat lung cancer [60].

Advancements in transcriptomics has led to the application of single-cell transcriptomics in precision medicine by comprehending the cellular diversity present in the tumor microenvironment and understanding cell–cell interaction in complicated heterogeneous malignant tissues. In a study, Kim et al*.* investigated the intra-tumoral heterogeneity of two primary renal cell carcinomas and corresponding lung metastases using scRNA-seq. Based on the anticipated activation of multiple drug target pathways, the authors developed a combinatorial regimen co-targeting two pathways mutually exclusive for the metastatic cancer cells thereby demonstrating the potential of scRNA-seq in the formulation of a treatment strategy [61]. Integration of scRNA-seq with 3-D in vitro tumor models has addressed the limitation of sample size and throughput. In fact, scientists have coupled DNA barcoding with microfluidic techniques for the creation of highly scalable systems in genome-wide scRNA-Seq [62, 63]. Another study integrated the 3-D cultures with scRNA-Seq to analyze the pancreatic ductal adenocarcinoma (PDAC) pathophysiology for drug screening and discovery [64] as well as opened potential paths for effective therapy discovery using laboratory-engineered glioblastoma (GBM) organoids (LEGO) [65]. More specifically, in the latter study, the authors utilized the pan-omics approach including the scRNA transcriptomics to not only characterize the LEGO but also define the genetic heterogeneity of the disease and various mesenchymal signatures. Using this approach, the authors demonstrated that glycerol lipid programming is a hallmark of GBM and LEGO and showed its potential for identifying novel molecular features and personalized treatment of GBM [65]. Hence, such high throughput sequencing systems can not only infer the potential drug targets but can also be valuable for interpreting the drug response.

Besides mRNAs, microRNAs (miRNAs) are commonly dysregulated in human cancers and play a critical role in the regulation of various genes [66–72]. MiRNAs are abundantly secreted through exosomes and can effectively mediate communication between cancer cells and normal cells, thereby contributing to cancer progression and metastasis [73, 74]. MiRNAs have been extensively profiled in 3-D in vitro tumor models; Nagai et al*.* performed a comprehensive analysis of miRNA profiles of colorectal cancer organoids (CRC) and colorectal adenoma (CRA). The authors found that exosomal miRNA signatures were differentially expressed in CRA and CRC organoids. More specifically, the expression of miR-1246 was higher in CRC-derived organoids than in CRA-derived organoids, suggesting the role of miR-1246 in cancer progression [75]. Another group compared the exosomes produced by 2-D cell culture and 3-D spheroids based on pancreatic cancer cell line, PANC-1. The authors demonstrated that exosomal miRNA and GPC-1 expression derived from spheroids showed more features pertinent to the progression of pancreatic cancer, and it was demonstrated that PANC-1 cells cultured in 3-D spheroids produced more exosomes than PANC-1 2-D cells. These results suggest the possible value of spheroids as an in vitro model for the investigation of cancer development and progression [76].

Taken together, transcriptomic analysis of the 3-D in vitro tumor models not only demonstrates great potential in identification of molecular subtypes, tumor heterogeneity and drug resistance but also predict response to chemotherapeutic agents thereby directing appropriate clinical decision making.

### Proteomics and 3-D in vitro tumor models

The study of all the proteins present in a cell, tissue, or organism under a certain, predetermined set of conditions is known as proteomics. Identification of unknown proteins in a sample relies on three fundamental techniques namely, fractionation of complicated protein or peptide mixtures, MS, and bioinformatics for processing and assembling the MS data [77]. Thus, multiple proteins within a sample can be quantitatively and qualitatively profiled using proteomics technique. The most effective method for acquiring high-resolution spectra of mixed peptides is liquid chromatography with tandem mass spectrometry (LC–MS/MS), which enables the identification of specific and sensitive biomarkers [78, 79]. The application of proteomics based on high throughput drug screening and the evaluation of therapeutic efficacy using 3-D in vitro tumor models such as PDOs is largely unexplored. Nevertheless, strong differences have been observed in the protein profiles of 2-D and 3-D cultures wherein the 3-D cultures demonstrated enhanced similarity to the in vivo/parent tumor [80]. In this regard, Buenafe et al*.* reported mass-spectrometric proteomic and functional protein network analysis of extracellular vesicles of pancreatic organoids; the authors revealed the involvement of vesicle proteins from healthy and pancreatic cancer organoids in cellular homeostasis and vesicular transport, respectively. In addition, the tumor-promoting markers, LAMA5, SDCBP, and TENA were found to be upregulated in PDAC vesicles, thereby demonstrating their potential as biomarkers for early disease diagnosis or disease and treatment monitoring [81]. Similarly, 3-D in vitro tumor models were characterized using proteomic studies and have demonstrated potential as drug screening platforms [9, 82]. Most recently, pan-omics analysis on commercially available colorectal cancer organoids revealed the role of SMAD4 inactivation in pro-migratory, cell proliferation and tumorigenesis. Interestingly, SMAD4-deficient CRC organoids secreted Dickkopf-related proteins, DKK3 and DKK4, that suppress the anti-tumor activity of natural killer cells. In addition, CRC patients with SMAD4 mutations and high expression of DKK4 exhibit poor prognosis thereby demonstrating new therapeutic approaches for advanced colorectal cancers [83]. Another study carried out baseline proteo-transcriptomic analysis in PDO lines from advanced colorectal cancer patients and showed that the activation of the transfer RNA (t-RNA) aminoacylation process and oxidative phosphorylation pathway was prominent in oxaliplatin non-responder PDOs. Furthermore, SWATH-mass spectrometry and RNA-seq methods were used to predict the treatment response or resistance thereby generating an effective platform for personalized medicine [84]. The proteome and phosphoproteome control a variety of cellular processes including DNA replication, apoptosis, and invasion; accordingly, several studies revealed variations in the proteome [85–88] and phosphoproteome [89, 90] between 2-D and 3-D culture models. For example, Yue et al*.* identified a total of 5867 protein groups, including 2523 phosphoproteins and 8733 phosphopeptides in colon carcinoma while comparing the HT29 2-D culture and 3-D spheroids and revealed their role in oxidative phosphorylation pathways, peroxisome pathways, metabolic pathways, and amino acid biosynthesis in 3-D cultures [90].

Tumor secretomes have also been explored as a source of biomarkers as well as therapeutic targets for potential management of cancer. In the context of 3-D in vitro tumor models, secretomes include cytokines, chemokines, and growth factors that are detected using immunoassays [91]. Multiplexed immunoassay-based beads have also been employed for determination of the concentrations of cytokine and growth factors in the supernatant of multicellular spheroid tricultures [92]. This involved 3-D co-cultures using tumor cells, fibroblasts and monocytes along with phenotypic profiling of the monocytes via expression analysis of cell surface markers and related soluble factors. The study further indicated that polarized monocyte-derived macrophages (MDMs) inhibited the in vitro activation and proliferation of CD4 + and CD8 + T cells, further demonstrating the immunosuppressive function of MDMs. The immunosuppressive effect was partially reversed when the 3-D co-cultured spheroids were treated with therapeutic molecules that further reactivated spheroid-polarized MDMs and showed the potential of 3-D models in drug testing applications [92]. Another study that was focused on the cytokine profile of a novel 3-D culture system to mimic immune checkpoint blockade (ICB) presented a novel method to screen the response of patient tumors toward ICB therapy. This method involved the evaluation of murine- and patient-derived organotypic tumor spheroids (MDOTS/PDOTS). The organotypic tumor spheroids not only mimicked the resistance and sensitivity to ICB therapy ex vivo but were also tested for new combinatorial therapies with PD-1 blockade. Moreover, the combination therapies including inhibitors TBK1, and CDK4/6, resulted in increased response to PD-1 blockade, both ex vivo and in vivo. In our viewpoint, this study demonstrates the potential of organotypic tumor spheroid as a platform for identifying and evaluating therapeutics and combinatorial therapies for ICB [93].

Taken together, the fusion of 3-D disease models and proteomics holds enormous untapped promises toward improved drug discovery. Since proteome is the primary functional component of cells and determines how they communicate with one another, it plays a major role in the onset and progression of the disease. However, further studies using proteomic techniques such as mass cytometry-based single-cell proteomics and spatial proteomics are warranted to fully explore the potential of proteomics and 3-D disease models in drug discovery [94, 95].

### Metabolomics and 3-D in vitro tumor models

Metabolomic techniques, including mass spectrometry and nuclear magnetic resonance (NMR) spectroscopy, have shown wide application in 3-D in vitro tumor model characterization, metabolomic profiling, and identification of therapeutic targets [96–103].

MS has been employed in the context of 3-D in vitro tumor models, to detect and quantify metabolites in distinct parts of the tumor and under different micro-environmental conditions [98]. As an example, metabolomics combined with matrix-assisted laser desorption/ionization mass spectrometry imaging (MALDI-MSI) was applied to investigate the effect of environmental pollutants on tumor progression. More specifically, significant changes in several metabolites, including ATP, ADP, and AMP were observed in breast cancer cell spheroids following exposure to a common environmental contaminant, bisphenol S (BPS). The MSI data further associated BPS-induced proliferative response with an increase in energy supply in the outer layer of tumor spheroids [98]. MALDI-MSI has also been used to study drug distribution patterns in patient-derived colon tumor organoids (CTOs) and to predict patient-specific treatment responses as well as personalized dosage regimens [102].

Compared to other OMICS technologies, metabolomic techniques allow the user to substantiate and validate the relevance of 3-D in vitro tumor models compared to 2-D cultures. For instance, a study by Rosi et al*.* revealed considerable changes in the lipid profile in breast cancer cells during various stages of cell growth in monolayer by proton NMR (^1^H NMR) spectroscopy. Interestingly, the 3-D in vitro tumor model spectra showed that lipid metabolism in 3-D spheroids resembles that of the confluent stage of monolayer cell cultures [104]. However, in another study, ^1^H NMR Spectroscopy demonstrated enhanced levels of mobile lipids, neutral lipids, and glutamine (Gln) signals in 3-D spheroids compared to 2-D monolayer culture, which emphasized the differences in metabolomic profile between the two culture types, before and after gamma irradiation [101]. Similarly, Lu et al*.* demonstrated the integration of 3-D co-culture model-based on leukemia and stromal cells for application in high-throughput/high-content metabolic drug screening using direct infusion mass spectrometry (DIMS) [97]. It is now clear that the method of cell culture can cause a significant change in the phospholipid profile and glucose accumulation as determined by ^1^H NMR spectrometry, even when the cells are derived from the same donor [100]. These studies also demonstrate that the 3-D in vitro tumor models are associated with physiologically relevant metabolic profiles, as well as with modulated treatment responses compared to 2-D monolayers.

Interestingly, the integration of metabolomics with 3-D in vitro tumor models has also been implemented in elucidating the process of disease progression and development [105, 106]. For example, significant intracellular and extracellular metabolic differences were seen in ovarian cancer cells (OCC) compared to spheroid-derived ovarian cancer stem cells (OCSC) using two-dimensional gas chromatography -mass spectrometry (GC x GC-MS). More specifically, arginine and proline metabolism pathways were found to behave differently in OCCs and OCSCs [105]. Another study identified metabolomic variations, mostly based on tricarboxylic acid cycle, amino acid metabolism, and glutamine between actively proliferating and quiescent mammary cells within an organotypic 3-D tissue culture. Additionally, the authors revealed that rapidly proliferating cells engage in anabolic carbon and nitrogen metabolism by boosting non-essential amino acid production and glutamine anaplerosis [106]. These findings underscore the importance of metabolomics in cancer research and emphasize how the metabolomic variations are crucial in identifying the intricate mechanism of actively proliferating cancer cells particularly with the help of in vitro 3-D tumor models.

Towards the identification of therapeutic targets, a recent study examined the metabolomic, transcriptomic and genomic profiles of triple-negative breast cancer (TNBC) patients particularly in transcriptomic basal-like immune-suppressed (BLIS) and luminal androgen receptor (LAR) tumors. Integration of these data with drug response studies using 3-D in vitro tumor models and mini patient-derived xenograft (mini-PDX) models led to the identification of sphingosine-1-phosphate (S1P), a ceramide pathway intermediate, and N-acetyl-aspartyl-glutamate (NAAG), a vital tumor-promoting metabolite in BLIS tumors, as viable targets for treatment of LAR tumors and high-risk BLIS tumors respectively [107]. Recently, a novel protocol for analyzing metabolic drug response in 3-D in vitro tumor models using liquid chromatography quadrupole time-of-flight mass spectrometry (LC-QTOF-MS) technique was established by Neef et al. [108]. Likewise, an integrated Biomimetic Array Chip (iBAC) enabling co-culture of 3-D liver and tumor tissues was used to assess drug-drug interactions and metabolism-induced anticancer bioactivity thereby supporting drug development. Further, prodrugs and their metabolites were determined using ultra- performance liquid chromatography coupled with mass spectrometry (UPLC-MS) technique [96].

Collectively, it is now evident that combining the power of high throughput omics techniques with 3-D in vitro tumor models enables an in-depth understanding of the metabolic variations that occur in cancer cells and their surrounding environment, and the findings can then be translated into the discovery and development of personalized cancer therapies.

### Lipidomics and 3-D in vitro tumor models

Within the solid tumors, cancer cells alter their fatty acid metabolism to adapt to impaired tumor environments, such as hypoxia and metabolic stress, and are associated with poor prognosis [109–111]. Accordingly, lipidomics is an important technology for drug screening and examining the lipid species that can contribute to key tumorigenic mechanisms as well as chemotherapeutic strategies [112]. Although lipidomics has rarely been used in 3-D in vitro tumor models, through comparative studies, it is now evident how 2-D drug screening could underestimate crucial metabolism inhibitors [113, 114]. In a study, the lipid contents and their spatial arrangement was determined by LC–MS and Raman chemical imaging respectively. The study found notable variations in the lipid makeup of both precancer and invasive spheroids, as well as in the surrounding cellular microenvironment when comparing 2-D and 3-D cell cultures [115]. In another study, LC–MS and GC–MS-based lipidomic analysis revealed significant alteration in complex lipid species, including ceramide and sphingomyelin, in a breast cancer 3-D in vitro tumor model [116]. In yet another study, the influence of differential lipid metabolism on therapeutic resistance in 2-D and 3-D systems was determined using GC–MS; the results demonstrated a significant correlation between the expression of stearoyl-CoA-desaturase (SCD1) and the progression of BRAF-mutated melanoma. Additionally, it was observed that 3-D cultures exhibited greater resistance to BRAF inhibitors compared to 2-D cultures [117]. In addition, another group studied the involvement of ascites in the metabolism and metastasis of ovarian cancer. While acidic environment inhibited the growth of ovarian cancer spheroids, alkaline ascites environment supported ovarian cancer progression. Metabolomics using ^1^H-NMR spectroscopy revealed the involvement of lipid metabolites that were linked with peritoneal pH, a parameter that plays an important role in the pathogenesis of ovarian cancer. The authors concluded that lipid metabolites, cytokines, chemokines, and physical–chemical characteristics could help in the stratification of ovarian cancer patients with malignant ascites [118].

Overall, these findings support the concept that 3-D in vitro tumor models could replicate the lipid microenvironment more precisely compared to 2-D models and could be utilized to examine the role of altered lipid profiles in cancer with the help of omics technology.

### Epi-omics and 3-D in vitro tumor models

Omics technologies can also be applied to study epigenetics to understand how epigenetic changes influence cancer initiation and progression. Chromatin immunoprecipitation with DNA sequencing (ChIP-seq), methylated DNA immunoprecipitation in combination with next-generation sequencing (MeDIP-seq), assay for transposase-accessible chromatin with NGS (ATAC-seq) and enhanced reduced representation bisulfite sequencing (ERRBS) are the various epigenomics techniques used to investigate the effect of epigenetic modifications and have been well established in cancer [119].

Epigenomics has been used to validate the 3-D in vitro tumor models to understand disease progression [120, 121] and for drug screening [121]. As an example, CpG-rich methylation analysis by ERRBS was used to study the changes in DNA methylation that occur in prostate cancer organoids, in comparison with the corresponding patient tissue sample. Prostate cancer organoids were also used to investigate the biological significance of the epigenetic modifier, EZH2, in controlling molecular pathways linked to neuroendocrine prostate cancer development [122]. Additionally, Berger et al*.* conducted an integrative study in prostate cancer focusing on the oncogenic transcription factor, N-Myc, using in vivo, in vitro, and PDO models. ChIP-seq/RNA-seq data revealed a significant, androgen-dependent alteration in the N-Myc cistrome, transcriptome, and histone methylation, which confirmed the role of N-Myc in the transcriptomic and epigenomic reprogramming of prostate cancer epithelial cells [120].

In an exciting study by Lin et al*.*, pan-omics analysis was applied to validate and characterize 3-D bio-printed osteosarcoma model [121]. With the integration of DNA methylomics and transcriptomics, the authors delineated the importance of autophagy in osteosarcoma. KEGG analyses of differential methylation positions (DMPs) in various tumor models highlighted that adherens and autophagy junction pathways were significantly altered in the 3-D in vitro tumor models further confirming that 3-D culture modulates the gene expression of different pathways via controlling epigenetic activity [121].

Epi-transcriptomics techniques like N6-methyladenosine (m^6^A) sequencing enable researchers to examine the modifications to RNA molecules and how they affect the onset and progression of cancer. In an interesting study, M^6^A methylated RNA immunoprecipitation sequencing (m^6^A MeRIP-seq) was employed to elucidate pathways and targets controlled by YTHDF1-m^6^A in CRC models. RhoA activator ARHGEF2 was found as a significant downstream target of YTHDF1 through integrative pan-omics analysis. The tumorigenic effects of this axis were also confirmed in CRC cell lines, 3-D organoids, and YTHDF1 transgenic mice [123].

**Fig. 2:**
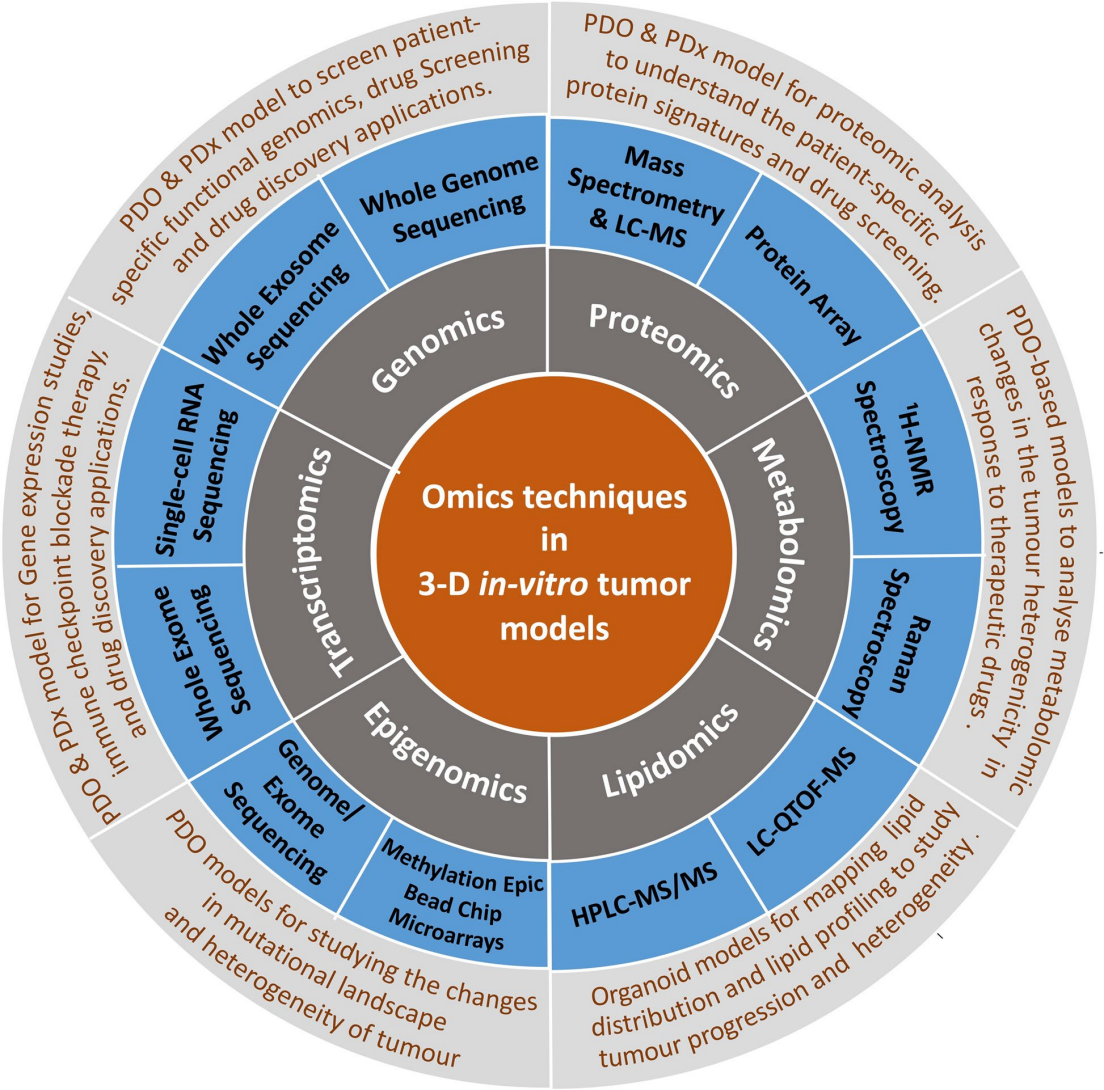
Omics techniques and their applications in 3-D in vitro tumor models. The figure presents a compilation of applications of pan-omics technology when integrated with 3-D in vitro tumor models for drug discovery and precision medicine. In the inner block arcs (blue and dark grey), various omics technologies, including whole genome sequencing, whole exome sequencing, microarray, LC–MS, NMR spectroscopy are enumerated. The outermost block arc (in light grey) illustrates the diverse applications of omics techniques, encompassing tumor model validation, tumor heterogeneity studies, the use of 3-D in vitro tumor models for drug screening and drug discovery platforms, as well as the identification of tumor protein signatures

In order to understand oxaliplatin drug response in 3-D CRC tumor models, integrated chromatin accessibility and transcriptomic profiling were carried out using ATAC-Seq and RNA-Seq methods. The results suggested significant alterations in chromatin opening in 28 genes following oxaliplatin treatment. Thus, the study elucidated the chromatin accessibility changes for genes that have a crucial role in oxaliplatin resistance [49]. Further, combining ChIP-seq and ATAC-seq has made it possible to analyze open chromatin sites and DNA/protein-binding sites using 3-D in vitro tumor models [124]. These studies highlight the progress in omics technologies for examining the vital role of epigenetics in tumorigenesis. In conclusion, 3-D in vitro tumor models offer a promising, clinically relevant drug screening/discovery platform by closely mimicking the interaction between tumor cells and the extracellular matrix compartment. Integrating the omics technology with these tumor models unravel the detailed mechanisms of interaction between cancer cells and stroma, in order to foster their validation and translational applications. Table 1 provides a succinct summary of selected studies performed using various omics techniques in 3-D in vitro tumor models. Figure 2 depicts the omics techniques and their applications in 3-D in vitro tumor models. The upcoming section discusses about how integration of the pan-omics technologies with these bioengineered 3-D in vitro tumor models supports tailored personalised treatments.

**Table 1:** Brief description of studies done using various omics techniques and 3-D in vitro tumor models

Sl. No	Type of omics used	Method adopted	Type of cancer studied	Purpose of the study	References
1.	**Genomics**	WGS	Breast cancer	Validation of PDO model using patient data	[125]
2.		WGS	Gastrointestinal cancer	Validation of PDO model using patient data	[126]
3.		WGS	Colorectal cancer	Validation of PDO model using patient data	[127]
4.		WGS	Esophageal cancer	Validation of PDO model using patient data	[128]
5.		WGS	Gastric cancer	Human and mouse gastric cancer organoids to check different drug targets	[129]
6.		NGS	Ovarian tumor & endometrial tumor	Validation of PDO model using patient data	[130]
7.		WGS	Gastroenteropancreatic (GEP) neuroendocrine neoplasm	Validation of PDO model for identification of new therapeutic targets	[37]
8.		WGS	Pancreatic cancer	Validation of 3-D models for identification of genomic markers contributing towards drug sensitivity	[39]
9.		WGS	Kidney cancer	Validation of PDO model based on pediatric kidney cancer patients	[38]
10.		WGS	Colon cancer, Breast cancer	PDO based model for studying the changes in mutational landscape of tumor when exposed to fluoropyrimidines	[131]
11.		WGS	Ovarian cancer	PDO based models to understand the inter- and intra-patient heterogeneity to drug response	[132]
12.		WES	Prostate cancer	PDO model to understand association of EZH2 driven molecular changes with cancer progression	[122]
13.		WES	Prostate cancer	Recapitulation of molecular diversity of the various subtypes of prostate cancer and validation of PDO Model	[36]
14.		WES	Gastric cancer	Establishment of primary gastric cancer organoid biobank & molecular profiling	[133]
15.		WES	Colorectal cancer	Biobank establishment, gene-drug association, validation using PDO model and 3-D model	[10, 35, 134]
16.		WES	Brain cancer	PDO models of pediatric high-grade gliomas to understand the role of hypoxia in cancer progression	[135]
17.		CRISPR-Cas9 screening with NGS	Colon cancer	PDO model to screen patient-specific functional genomics	[41]
18.	**Transcriptomics**	Microarray	Breast cancer	Studied the gene expression profile affecting the drug response in 3-D compared to 2-D model	[23, 24]
19.		RNA-Seq	Colorectal cancer	Transcriptomic and chromatin profiling for personalized drug targets to overcome chemoresistance	[49]
20.		RNA-Seq	Endometrial cancer	PDO based Biobank development which recapitulated the lesions from all clinical stages and can be used for drug screening purposes	[50]
21.		RNA-Seq	Breast cancer	PDO model validation using patient data	[125]
22.		RNA-Seq	Pancreatic cancer	PDO & PDX derived organoid model for understanding genomic and histopathological changes along with drug testing	[136]
23.		RNA-Seq	Colorectal cancer	Colonic organoids from induced pluripotent stem cells used in disease modelling and drug discovery for colorectal disease	[137]
24.		RNA-Seq	Bladder cancer	Validation of PDO model using patient data	[58, 138]
25.		RNA-Seq	Liver cancer	Micro-scaffold-based model to enumerate relationship between EMT status and hepatic functions	[139]
26.		RNA-Seq	Brain cancer	PDOs developed via CRISPR-Cas9-mediated mutagenesis for drug testing	[140]
27.		RNA-Seq	Colorectal cancer	Patient-derived organotypic tumor spheroids in 3-D microfluidic culture to screen the response of immune check point blockade therapy	[93]
28.		RNA-Seq	Colorectal cancer	Model validation of 2-D patient derived cancer cells, 3-D air-liquid interface based organoid culture and xenograft for identification of potential drug targets	[141]
29.		RNA-Seq	Colorectal cancer	PDOs for therapeutic drug screening	[54]
30.		RNA-Seq	Lung cancer	Development of PDO models to recapitulate the tumor heterogeneity and to identify the targets against Wnt signalling	[60]
31.		RNA-Seq	Pancreatic cancer	PDO based models to identify transcriptomic groups involved in invasion	[64]
32.		RNA-Seq	Brain tumor	Pediatric patients derived 3-D culture model to study the microenvironment induced gene expression changes	[142]
33.		GeneChip™ Human Transcriptome Array	Metastatic colorectal cancer	Pharmacogenomic profiling for PDO models to check the drug sensitivity	[59]
34.		Single‐cell RNA-Seq	Metastatic colorectal cancer	PDO formed to recapitulate the tumor heterogeneity and further used to study the drug response	[52]
35.		Single‐cell RNA-Seq	Metastatic renal cell carcinoma	3-D model for combinatorial drug therapy	[61]
36.		Single‐cell RNA-Seq	Glioblastoma	Laboratory engineered glioblastoma organoid models to recapitulate genetic heterogeneity and identification of various drug targets	[65]
37.		Single‐cell RNA-Seq	Multi organ tumors	PDO based models to recapitulate tumor immune microenvironment for personalized therapeutic applications	[143]
38.		miRNA Microarrays	Colorectal adenoma and colorectal cancer	PDO utilised to understand the miRNA signature patterns in colorectal adenoma and colorectal cancer	[75]
39.	**Proteomics**	Mass spectrometry	Pancreatic cancer	Extracellular vesicle protein profiling to investigate tumorigenesis	[81]
40.		Mass spectrometry	Colorectal cancer	Organoid based model to show the effect of SMAD4 inactivation on metastasis	[83]
41.		Mass spectrometry	Pancreatic cancer	Murine and human derived organoids to investigate the pancreatic cancer pathogenesis	[144]
42.		Mass spectrometry	Colorectal cancer	Organoids from healthy donor and cancer patients subjected to proteomic analysis to understand the patient-specific protein signatures	[145]
43.		DigiWest multiplex protein profiling	Colorectal cancer	PDO based model for studying tumor heterogeneity and prediction of therapy response	[146]
44.		Single-cell mass cytometry (CyTOF®)	Breast cancer	Primary breast organoids to preserve complex epithelial lineage	[147]
45.		LC-MS/MS analysis	Brain cancer	Drug screening platform using patient derived neurospheres/3-D culture	[85, 148]
46.		LC–MS/MS -SWATH	Colorectal cancer	PDO based model to integrate the drug sensitivity with proteomics for prediction of better therapeutic response	[84]
47.		LC-MS/MS analysis	Colon cancer	Quantitative proteomic and phosphoproteomic analysis of 3-D spheroids	[90]
48.		Reverse phase protein microarray	Colon cancer	PDO based model to study altered molecular pathway involved in tumorigenesis	[33]
49.		Reverse phase protein microarray	Multiple Cancers	Compared 121 different phosphorylated and non-phosphorylated proteins between 2-D and 3-D cell cultured models under the influence of hypoxia	[89]
50.		Protein Array	Colorectal cancer	PDO based models to identify the role of MIR21 dysregulation, JAM-A silencing and signalling pathways involved in tumorigenesis	[149]
51.		Milliplex® MAP Human Cytokine/Chemokine Panel	Pancreatic cancer	To study the effect of tumor cells and fibroblast on monocytes in a 3-D co-culture model	[92]
52.	**Metabolomics and lipidomics**	^1^H-NMR Spectroscopy	Brain cancer	To validate patient-derived model in hypoxic microenvironment	[135]
53.		Mass spectrometry and Raman chemical imaging	Breast cancer	Cell line-based spheroid model to map lipid distribution during disease progression	[115]
54.		Mass spectrometry	Breast cancer	Spheroid based model to study the effect of bisphenol- S on tumor progression	[98]
55.		Mass spectrometry	Prostate and breast cancer	Spheroid models to evaluate the alterations in intracellular lipid concentrations when treated with metabolic enzyme inhibitors	[113]
56.		LC-QTOF-MS	Colorectal cancer	PDO based models used for metabolomic and lipidomic profiling when treated with 5-fluorouracil	[108]
57.		Optical metabolic Imaging	Breast cancer and pancreatic cancer	PDO based models to analyse the optical metabolic imaging of cellular heterogeneity as a predictor of clinical treatment response	[150]
58.		LCMS	Breast cancer	PDO based model to describe the metabolomic landscape of TNBC patients	[107]
59.		HPLC-MS/MS	Colorectal cancer	Understanding the mechanism of curcumin in PDO based model	[151]
60.		Organ on chip platform with electrochemical microsensors	Breast cancer	Patient derived breast cancer stem cell based organoid model for in situ metabolite monitoring and drug screening	[152]
61.		Mass spectrometry	Ovarian cancer	PDO based model for drug testing and to evaluate the role of lipid metabolic activities in cancer progression	[112]
62.		UHPLC	Colorectal cancer	PDO based models to identify the role of drug sensitizing activity of spirulina polysaccharides in 5 fluorouracil resistant CRC organoids	[153]
63.		UPLC-MS	Multiple tumor cells	Chip based 3-D co-culture model to evaluate metabolism induced anticancer activity	[96]
64.		MALDI Mass Spectrometry Imaging	Colorectal cancer	PDO based model to examine drug and its metabolite distribution	[102]
65.		GCxGC-MS Analysis	Ovarian cancer	To evaluate the difference in intracellular and extracellular metabolite profile amongst ovarian cancer cell and spheroid derived ovarian cancer stem cells	[105]
66.		LCMS	Breast cancer	To understand the changes in lipidome during breast cancer metastasis	[116]
67.	**Epigenomics**	DNA methylation profiling	Kidney cancer	Validation of PDO model based on pediatric kidney cancer patients	[38]
68.		Enhanced reduced representation bisulfite sequencing	Prostate cancer	PDO based models used to understand the role of epigenetic modifier EZH2 in cancer progression	[122]
69.		ATAC Seq	Pancreatic cancer	PDO based model to study chromatin accessibility associated with drug sensitivity	[154]
70.		Whole-genome bisulfite sequencing	Colorectal cancer	PDO based model used to evaluate the transcriptomic and epigenetic landscape with respect to the culture conditions	[155]
71.		Methylation epic bead ChIP microarrays	Colorectal cancer	PDO based model used to prove the anti-metastatic activity of ginseng by inhibiting the expression of DNA methyltransferases	[156]
72.		ChIP Seq	Prostate cancer	PDX/PDO models to understand the lineage plasticity and epigenetic reprogramming induced by N-Myc	[120]
73.		Methylation array analysis	Osteosarcoma	Comparing 3-D bio-printed model with other tumor models for changes in cell cycle, metabolomics and epigenetic regulation	[121]
74.		m6A methylated RNA immunoprecipitation sequencing	Colorectal cancer	The tumorigenic effect of YTHDF1-m6A-ARHGEF2 axis on disease progression studied on organoids and mice models	[123]

*WGS* whole genome sequencing, *WES* whole exome sequencing, *PDOs* patient-derived organoids, *PDX* patient-derived xenografts, *CRC* colorectal cancer, *LC-MS/MS* liquid chromatography-tandem mass spectrometry, *MIR21* MicroRNA-21, *JAM-A* junctional adhesion molecule-A, ^1^*H-NMR* proton nuclear magnetic resonance, *LC-QTOF-MS* liquid chromatography quadrupole time-of-flight mass spectrometry, *HPLC-MS/MS* high-performance liquid chromatography mass spectrometry, *NGS* next generation sequencing, *RNA-Seq* RNA sequencing, *UHPLC* Ultra high-performance liquid chromatography, *UPLC-MS* Ultra-performance liquid chromatography coupled with mass spectrometry, *MALDI MSI* Matrix-assisted laser desorption/ionization – Mass spectrometry imaging, *GCxGC-MS* two-dimensional gas chromatography coupled to mass spectrometry, ChIP- chromatin immunoprecipitation, *CyTOF®* cytometry by Time-Of-Flight

## Omics in precision medicine: focus on 3-D in vitro tumor models

The goal of precision medicine is to offer clinical benefits to individuals based on their unique molecular matches [157]. Genotyping and genomics have significantly influenced the treatment modalities and management of several malignancies, including breast and ovarian cancer [158]. Numerous studies have demonstrated that NGS-based approaches provide genetic information such as mutational status and gene expression patterns, from tumor and stromal cells of the tissues from cancer patients. This genetic information enables sequence-matching therapy, which could eventually enhance the overall drug response and survival rates of cancer patients [159]. Despite the advancements in the development, establishment, and application of 3-D in vitro tumor models, these technologies have not yet made a significant impact on cancer clinical trials for tailored drug screening. Nevertheless, there are numerous PDO and PDX models that are undergoing feasibility studies (NCT03453307, NCT03544255, NCT03577808, NCT03655015, NCT03890614, NCT03979170, NCT03990675, NCT04261192, NCT04371198) demonstrating that these technologies are still advancing in the field of clinical functional medicine. More specifically, the EXALT study was the first prospective trial in precision medicine that used a tailored functional medicine assessment to direct treatment choice. This study tested a treatment for individuals with advanced, aggressive hematological cancers using an image-based single-cell drug screening technology with automated high-content microscopy and image analysis. The authors demonstrated that the progression-free survival (PFS) was improved by 1.3-fold for 54% of patients who were treated using single-cell functional precision medicine compared to earlier therapies. About 40% of these patients had exceptional responses that lasted three times longer than expected for their condition [160]. In conclusion, similar platforms based on PDOs and PDXs can more faithfully recreate the disease state and patient response to various therapies.

In yet another study, Seppälä et al*.* generated pancreatic cancer PDOs and analyzed them using NGS and pharmacotyping. PDO-specific pharmacotype was evaluated prospectively, via a randomized controlled clinical trial, as a predictive indicator of clinical treatment response. This study successfully predicted the clinical treatment response in locally advanced pancreatic cancer by ex vivo PDO pharmacotyping with FOLFIRINOX components and the patients were treated using an induction or neoadjuvant approach [161]. While combination therapies have demonstrated high effectiveness in cancer patients, they are often associated with serious adverse effects thereby demanding administration of lower doses or cycle counts of treatment. Hennig et al*.* determined whether PDOs could be used to tailor poly-chemotherapy regimens, including neoadjuvant and adjuvant chemotherapy in pancreatic ductal adenocarcinoma. Pharmacotyping of chemotherapy-naive patients and neoadjuvant-treated PDOs demonstrated the potential of PDOs in establishing personalized poly-chemotherapy strategies [162]. Moreover, 3-D in vitro tumor models based on PDOs have also been implemented in clinical trials and most recent results from SWOG-S1505 trial (NCT02562716) demonstrated equal effectiveness of FOLFIRINOX or Gem/nab-Pac regimen when used as neoadjuvant therapy [163, 164]. The results of these studies suggest that 3-D in vitro tumor models including PDOs can potentially support the clinical decision making of treatment regimen and can also be extrapolated for drug discovery.

## Pan-omics repositories and bioinformatic tools: focus on 3-D in vitro tumor models

The exponential rise in omics databases has led to the emergence of the ‘Big Data’ concept in the field of drug discovery and precision medicine.

The ‘Big Data’ data is an outcome of large-scale cancer genomics initiatives projects across the globe and its content can be categorized as molecular data (which includes gene expression, genomics, mutational and proteomics data), phenotypic data (obtained because of cellular perturbation), radiological imaging data, inter-omics interaction data, and textual clinical data [165–169]. In this regard, TCGA has generated a plethora of pan-omics data, including genomic, transcriptomic, epigenetic, and proteomic data; TCGA has been utilized by various researchers incorporating patient-derived 3-D in vitro tumor models/tumor organoids for drug discovery and precision medicine. As an example, Cho et al*.* developed a patient-derived colorectal cancer organoid (CCO) model and carried out the pairwise whole transcriptome sequence analysis of 87 CCOs and their matched primary tumors using the TCGA RNAseq pipeline [31].

A study by Mittal et al*.* introduced an innovative approach for the long-term evaluation of new drug combinations to address drug resistance in cancer [170]. For this purpose, the authors utilized cBioportal to conduct survival analysis for breast cancer patients with altered Insulin-like Growth Factor 1 Receptor (IGF1R) signalling pathway. The findings demonstrated an inverse relationship between IGF1 signalling and overall survival of both basal and HER2 + breast tumors. The authors evaluated the efficacy of combination therapy based on IGF1R inhibitor with chemotherapy such as paclitaxel and a HER2 inhibitor on 3-D organoid models developed by co-culturing cancer cells with endothelial cells; combination treatment led to a lower area under the curve (AUC) compared to individual treatments. Therefore, integration of the novel organoid method with the online data portals enables the simulation of more realistic culture conditions and the identification of potential therapeutic targets for further evaluation in clinical trials thereby leading to improved patient outcomes [170].

Additionally, TCGA databases have been combined with 3-D in vitro models to identify molecular subtypes of breast cancer and have effectively predicted the therapeutic response of breast cancer patients with various medications based on these subtypes [171]. The authors also developed a biobank, breast cancer PTDX encyclopedia (BCaPE) and demonstrates potential in pre-clinical breast cancer pharmacogenomic studies. TCGA database was also applied to identify subtypes of colorectal cancer using consensus molecular colorectal cancer classifier with the potential to establish 3-D tumor organoid platform as a tool to investigate the molecular mechanisms involved in pre-existing drug resistance and tumorigenicity in cancer cells. By integrating the TCGA data into a 3-D in vitro tumor model, the researchers aimed to enhance its capability to elucidate the molecular underpinnings of drug resistance and tumorigenicity in cancer [141].

In yet another study, integration of omics data from TCGA database and 3-D tumor models was exploited for discovery of novel prognostic markers. Broutier et al. conducted a study based on a systematic comparison of transcriptional differences between healthy organoid lines and primary liver cancer (PLC) organoid lines and identified markers associated with poor prognosis in hepatocellular carcinoma and cholangiocarcinoma with the help of TCGA database [172].

Apart from TCGA database, Gene Expression Omnibus (GEO), contains a wide range of high-throughput datasets related to various aspects of gene expression, such as RNA sequencing, GWAS, and epigenetic data, and has been combined with organoids and omics-based studies. As an example, Pranav et al. reviewed the significance of expressions of key genes in organoid cultures obtained from breast cancer patient-derived biopsies and utilized data from the GEO database to analyze the gene expression profile in breast cancer organoids [173].

There are other databases such as The Human Genome Variation Society (HGVbase), International Cancer Genome Consortium (ICGC), The Genomic Data Commons (GDC), and Human Cell Atlas (HCA) that can provide molecular profiles of various cancer types. In addition, a resource tool from the National Cancer Institute, Clinical Proteomic Tumour Analysis Consortium (CPTAC), performed proteogenomic studies in more than 1000 tumors in 10 cancer cohorts; they further integrated multi-omics datasets with clinical data to correlate genomic abnormalities and tumor characteristics thereby aiding in improved drug discovery, cancer diagnosis and treatment [174]. The application of these database deserves to be explored for the identification of novel targets using 3-D in vitro tumor models.

In conclusion, the integration and analysis of pan-omics data is a critical component of modern cancer research. By providing a more complete picture of the underlying biology of cancer, pan-omics data are enabling the development of new therapeutic approaches and advancing the field of personalized medicine. These platforms and databases assist researchers and clinicians by offering information and tools that enable them to understand the molecular alterations that occur in cancer cells. Decisions regarding the diagnosis and treatment of diseases can be made more effectively by incorporating this knowledge with data from 3-D in vitro tumor models. The combination of bioinformatics tools, disease modeling, and omics technologies will help in the development of more accurate predictive models, as well as the identification of new cancer targets and the comprehension of cancer heterogeneity. A summary of the databases and the analysis portals that host these data are described and enlisted in Supplementary Table S1.

## Pan-omics and 3-D in vitro tumor models: advantages and limitations

 Integration of omics technologies using 3-D patient derived in vitro tumor models has led to the identification of novel drug targets, enumerating the course of tumor progression using pan-omics technologies. In comparison to the PDX models, 3-D in vitro tumor models are associated with reduced turnaround time, and they can easily recapitulate the complexities of in vivo tumors with better prediction of therapy sensitivity [175]. Thus, by using limited starting sample, patient derived 3-D tumor models can be effectively developed and used for molecular profiling or to identify new therapeutic targets and development of biobanks. More specifically, multi-omics platforms have been applied for identification of genomic drivers that contribute to drug sensitivity and therapeutic response in 3-D in vitro tumor models [23, 40, 84, 176]. The 3-D in vitro tumor models have also contributed towards the identification of therapies and for appropriate clinical decision making for targeted therapies. In this regard, 3-D in vitro tumor models can be used as versatile high throughput platforms for drug testing and to study synthetic lethal therapies based on PARPi [46, 177]. In addition, these 3-D in vitro tumor models retain the intra-tumoral heterogeneity that can be used to elucidate dynamic cancer phenotypes and accordingly modulate therapeutic strategies. The advent of 3-D in vitro tumor model has also led to analysis of secretomes using omics for elucidation of ECM and exosome components associated invasion and organ-specific metastasis thereby leading to identification of potential therapeutic approaches aimed at targeting tumor stroma. In addition, ex vivo pharmacogenomic profiling on 3-D in vitro tumor models can be used to predict clinical response and associated mechanistic profiling.

Although amalgamation of pan-omics technologies and 3-D in vitro tumor models has revealed some interesting and exciting advancements in the field of drug discovery and personalized medicine, there are still some barriers for their widespread application to predict treatment responses. Towards this, the isolation of different cellular components according to the requirements of various omics technologies requires optimal harvesting and digestion protocols to obtain high cell yield and viability to obtain a representative dataset. This isolation step for various omics techniques relies on the initial patient sample.

In addition, other components present in the tumor model, such as biological remains of the scaffolds, extracellular matrix, and the presence of other cell populations in the multi-culture system, can further complicate the isolation of the desired cells from the 3-D model. Thus, obtaining an optimal sample required by the desired omics technology involves the selection of digestion and dissociation protocols. These protocols must be curated and optimized for each type of 3-D tumor model system. Other challenges include limited access to high-end technologies used for pan-omics analysis as well as the development of 3-D in vitro tumor models. Apart from this, sample preparation, experimentation, and analysis involved in pan-omics can be expensive, thereby demonstrating the importance of every research question asked in such experiments. In addition, the raw data available through pan-omics demands the involvement of bioinformatics experts and computational biologists who can generate meaningful information through the use of appropriate software and tools.

Furthermore, various chemokines, cytokines and growth factors released by different components of the tumor microenvironment such as CAFs, endothelial cells, platelets and immune cells influence the infiltration of immune cells such as cytotoxic T cells, NK cells, CD4 + T cells, macrophages and dendritic cells [15, 178]. However, there are limited studies on assessing the effect of these factors in 3-D in vitro tumor models. Even though tumor models find application in predicting the drug response, there are limited studies elucidating the screening of immune check points inhibitors, crosstalk with the immune system and vasculature using 3-D in vitro tumor models. In addition, while the neuroendocrine system and neurotransmitters also influence the anti-cancer potential of immunotherapy and chemotherapeutic drugs [179], there are limited reports evaluating the neurotransmitters or neuroendocrine system on drug efficacy using 3-D in vitro tumor model system.

Nevertheless, the combination of pan-omics technologies and 3-D in vitro tumor models has led to clinically relevant solutions for improved patient care.

## Conclusions and future perspectives

The heterogeneous nature of cancer has always been a major challenge in deciding the treatment modality for patients. The conventional treatment approach is usually impeded by the crosstalk between the native neoplastic cells and the surrounding tumor microenvironment, a phenomenon clinically called as intra-tumor or inter-tumor heterogeneity. With the advent of modern medicine, the focus is on the stratification of cancer patients based on disease subtypes, risk factors, clinical features, and demographic details, and then tailoring the line of treatment for the individuals using omics technology to maximize the benefit and limit drug-induced side effects [169].

Omics technology represents a remarkable platform for the identification of novel markers for the prognosis, diagnosis, and therapeutics of cancer. Various 3-D in vitro tumor model systems including PDOs recapitulate the heterogeneity of the primary tissue and thus represent as powerful tools for cancer research [180]. Further, the amalgamation of 3-D in vitro tumor models with pan-omics technologies has led to a paradigm shift in the field of drug discovery and precision medicine. These models have not only been validated for genotypic and phenotypic recapitulation of in vivo patient tumors but have also been utilized for therapeutic target identification, markers involved in chemoresistance, analysis of drug action, and understanding the pathophysiology of cancer progression. Therefore, omics-based discovery of the specific aspects of tumor microenvironment including immune system, vasculature, ECM and other stromal population within the 3-D in vitro tumor model can not only identify new therapeutic targets but also aid in precision therapy. In addition, understanding the effect of the microbiome, particularly in colorectal cancer, gastric cancer, oral cancer and cervical cancer-based 3-D models, on drug response can help design effective therapeutic strategies. The multi-omics approach focusing on the identification of somatic mutations in 3-D patient-derived in vitro tumor models can provide novel perspectives toward understanding the tumor microenvironment-associated chemoresistance. While this field is relatively new and demands technical and logistic support including the availability of high-end infrastructure, trained personnel, and reduction in costs, the outlook on the future applications of these technologies in drug discovery and precision medicine for improved management of cancer is enormously promising.

### Supplementary Information

**Additional file 1:** **Supplementary Table S1.** Cancer databases and portals used for the analysis of pan-omics data.

## Data Availability

Not applicable.

## Abbreviations

^1^H NMR: Proton NMR
2-D: Two-dimensional
3-D: Three-dimensional
ADP: Adenosine diphosphate
ALI: Air–liquid interface
AMP: Adenosine monophosphate
APC/KRAS: Adenomatous polyposis coli/ Kristen-ras
ARHGEF2: Rho guanine nucleotide exchange factor 2
ATAC- Seq: Assay for transposase-accessible chromatin with NGS
ATP: Adenosine triphosphate
AUC: Area under the curve
BLIS: Basal-like immune-suppressed
BPS: Bisphenol S
BRAF: V-raf murine sarcoma viral oncogene homolog B1
BRCA1/ BRCA2 gene: Breast Cancer gene 1/2
CAFs: Cancer-associated Fibroblasts
CC: Cholangiocarcinoma
cccDNA: Covalently closed circular DNA
CCO: Colorectal cancer organoids
CD4+: Clusters of differentiation 4
CD8+: Cluster of differentiation 8
CDK4: Cyclin dependent kinase 4
CDK6: Cyclin dependent kinase 6
CDx: Companion diagnostic tests
ChIP-seq: Chromatin immunoprecipitation sequencing
CPTAC: Clinical Proteomic Tumour Analysis Consortium
CRA: Colorectal adenoma
CRC: Colorectal cancer organoids
CRISPR: Clustered regularly interspaced short palindromic repeats
CTO: Colon tumor organoids
CyTOF®: Cytometry by Time-Of-Flight
DIMS: Direct infusion mass spectrometry
DKK4: Dickkopf WNT signaling pathway inhibitor 4
DMP: Differential methylation positions
ECM: Extracellular matrix
EGFR: Epidermal growth factor receptor
EMT: Epithelial-mesenchymal transition
ERBB3: Erb-B2 receptor tyrosine kinase 3
ERRBS: Enhanced reduced representation bisulfite sequencing
EZH2: Enhancer of zeste homolog 2
FASN: Fatty acid synthase
FFPE: Formalin-fixed, paraffin-embedded
FOLFIRINOX: F – fluorouracil (also called 5FU); Irin – irinotecan; Ox – oxaliplatin
GBM: Glioblastoma
GC: Gas chromatography
GC x GC-MS: Two-dimensional gas chromatography -mass spectrometry
GDC: Genomic Data Commons
GEO: Gene Expression Omnibus
GEP: Gastro-entero-pancreatic
GPCRs: G protein-coupled receptors
GWASs: Genome-wide association studies
HBV: Hepatitis B virus
HCA: Human Cell Atlas
HCC: Hepatocellular carcinoma
HER2: Human epidermal growth factor receptor 2
HGP: Human Genome Project
HGVbase: Human Genome Variation Society
HR: Homologous recombination
iBAC: Integrated biomimetic array chip
ICB: Immune checkpoint blockade
ICGC: International Cancer Genome Consortium
IGF1R: Insulin-like growth factor 1 receptor
IGFBP5: Insulin-like growth factor binding protein 5
KEGG: Kyoto Encyclopedia of Genes and Genomes
LAMA5: Laminin subunit alpha 5
LAR: Luminal androgen receptor
LC-QTOF-MS: Liquid chromatography quadrupole time-of-flight mass spectrometry
LC–MS/MS: Liquid chromatography with tandem mass spectrometry
LEGO: Laboratory engineered glioblastoma organoids
m6A MeRIP-seq: M6A methylated RNA immunoprecipitation sequencing
MALDI-MSI: Matrix-assisted laser desorption/ionization mass spectrometry imaging
MDM2: Murine double minute 2
MDMs: Monocyte derived macrophages
MDOTS: Murine derived organotypic tumor spheroids
MeDIP: Methylated DNA immunoprecipitation
miRNAs: MicroRNAs
MS: Mass spectrometry
MYCN: MYCN proto-oncogene
NAAG: N-acetyl-aspartyl-glutamate
NGS: Next-generation sequencing
NK cells: Natural Killer Cells
NKX2-1: NK2 homeobox 1
NMR: Nuclear magnetic resonance
OCC: Ovarian cancer cells
OCSC: Ovarian cancer stem cells
PANC-1: Pancreatic cancer cell line
PARP: Poly (ADP-Ribose) Polymerase
PD-1: Programmed cell death protein 1
PDAC: Pancreatic ductal adenocarcinoma
PDCCs: Patient-derived colorectal cancer cells
PDO: Patient-derived organoids
PDTO: Patient-derived tumor organoids
PDX: Patient-derived xenograft
PHH: Primary human hepatocyte
PIK3CA: Phosphatidylinositol-4,5-bisphosphate 3-kinase catalytic subunit alpha
PLC: Primary liver cancer
RAS: Rat sarcoma virus
RhoA: Ras homolog family member A
RNA-seq: RNA sequencing
S1P: Sphingosine-1-phosphate
scRNA-seq: Single-cell RNA-sequencing
SDCBP: Syndecan binding protein
SMAD4: Suppressor of mothers against decapentaplegic homolog 4
t-RNA: Transfer RNA
TBK1: TANK binding kinase 1
TCGA: The Cancer Genome Atlas
TENA: Tenascin C gene
TGFβ: Transforming growth factor-beta1
TGS: Third-generation of long reads sequencing
TIME: Tumor immune microenvironment
TNBC: Triple-negative breast cancer
TP53: Tumor protein 53
UHPLC: Ultra high-performance liquid chromatography
UPLC: Ultra-performance liquid chromatography
VEGF: Vascular endothelial growth factor
WES: Whole exome sequencing
WGS: Whole genome sequencing
Wnt: Wingless-related integration site
YTHDF1: YTH N6-methyladenosine RNA binding protein F1

## References

[1] Sung H, Ferlay J, Siegel RL, Laversanne M, Soerjomataram I, Jemal A (2021). Global Cancer Statistics 2020: GLOBOCAN estimates of incidence and mortality worldwide for 36 cancers in 185 countries. CA Cancer J Clin.

[2] Mohs RC, Greig NH (2017). Drug discovery and development: role of basic biological research. Alzheimers Dement (N Y).

[3] Kapałczyńska M, Kolenda T, Przybyła W, Zajączkowska M, Teresiak A, Filas V (2018). 2D and 3D cell cultures – a comparison of different types of cancer cell cultures. Arch Med Sci.

[4] Jensen C, Teng Y (2020). Is it time to start transitioning from 2D to 3D cell culture?. Front Mol Biosci.

[5] Barbosa MAG, Xavier CPR, Pereira RF, Petrikaitė V, Vasconcelos MH (2022). 3D cell culture models as recapitulators of the tumor microenvironment for the screening of anti-cancer drugs. Cancers (Basel).

[6] Arya N, Sardana V, Saxena M, Rangarajan A, Katti DS (2012). Recapitulating tumour microenvironment in chitosan–gelatin three-dimensional scaffolds: an improved in vitro tumour model. J R Soc Interface..

[7] Sayyed AA, Gondaliya P, Mali M, Pawar A, Bhat P, Khairnar A, Arya N, Kalia K (2021). MiR-155 inhibitor-laden exosomes reverse resistance to cisplatin in a 3D tumor spheroid and xenograft model of oral cancer. Mol Pharm.

[8] Pieters VM, Co IL, Wu NC, McGuigan AP (2021). Applications of omics technologies for three-dimensional in vitro disease models. Tissue Eng Part C.

[9] Driehuis E, Van Hoeck A, Moore K, Kolders S, Francies HE, Gulersonmez MC (2019). Pancreatic cancer organoids recapitulate disease and allow personalized drug screening. Proc Natl Acad Sci U S A.

[10] Pauli C, Hopkins BD, Prandi D, Shaw R, Fedrizzi T, Sboner A (2017). Personalized in vitro and in vivo cancer models to guide precision medicine. Cancer Discov.

[11] Gerber DE, Minna JD (2010). ALK inhibition for non-small cell lung cancer: from discovery to therapy in record time. Cancer Cell.

[12] Matthews H, Hanison J, Nirmalan N (2016). “Omics”-informed drug and biomarker discovery: opportunities, challenges and future perspectives. Proteomes.

[13] Paananen J, Fortino V (2020). An omics perspective on drug target discovery platforms. Brief Bioinform.

[14] Napoli GC, Figg WD, Chau CH (2022). Functional drug screening in the era of precision medicine. Front Med (Lausanne).

[15] Rodrigues J, Heinrich MA, Teixeira LM, Prakash J (2021). 3D In Vitro Model (R)evolution: unveiling tumor-stroma interactions. Trends Cancer.

[16] Hanahan D, Coussens LM (2012). Accessories to the crime: functions of cells recruited to the tumor microenvironment. Cancer Cell.

[17] Ubink I, Bolhaqueiro ACF, Elias SG, Raats DAE, Constantinides A, Peters NA (2019). Organoids from colorectal peritoneal metastases as a platform for improving hyperthermic intraperitoneal chemotherapy. Br J Surg.

[18] Dai X, Shen L (2022). Advances and trends in omics technology development. Front Med (Lausanne).

[19] McGinn S, Gut IG (2013). DNA sequencing - spanning the generations. N Biotechnol.

[20] The Cancer Genome Atlas Program – NCI. Accessed 2023 Jan 23. https://www.cancer.gov/about-nci/organization/ccg/research/structural-genomics/tcga

[21] Kaliyappan K, Palanisamy M, Govindarajan R, Duraiyan J (2012). Microarray and its applications. J Pharm Bioallied Sci.

[22] Hudson EA, Fox LH, Luckett JCA, Manson MM (2006). Ex vivo cancer chemoprevention research possibilities. Environ Toxicol Pharmacol.

[23] Horning JL, Sahoo SK, Vijayaraghavalu S, Dimitrijevic S, Vasir JK, Jain TK (2008). 3-D tumor model for in vitro evaluation of anticancer drugs. Mol Pharm.

[24] DelNero P, Lane M, Verbridge SS, Kwee B, Kermani P, Hempstead B (2015). 3D culture broadly regulates tumor cell hypoxia response and angiogenesis via pro-inflammatory pathways. Biomaterials.

[25] Ma L, Zhang B, Zhou C, Li Y, Li B, Yu M (2018). The comparison genomics analysis with glioblastoma multiforme (GBM) cells under 3D and 2D cell culture conditions. Colloids Surf B Biointerfaces.

[26] Sogawa C, Eguchi T, Namba Y, Okusha Y, Aoyama E, Ohyama K (2021). Gel-free 3d tumoroids with stem cell properties modeling drug resistance to cisplatin and imatinib in metastatic colorectal cancer. Cells.

[27] Naruse M, Ochiai M, Sekine S, Taniguchi H, Yoshida T, Ichikawa H (2021). Re-expression of REG family and DUOXs genes in CRC organoids by co-culturing with CAFs. Sci Rep.

[28] Gangapuram M, Mazzio EA, Redda KK, Soliman KFA (2021). Transcriptome profile analysis of triple-negative breast cancer cells in response to a novel cytostatic tetrahydroisoquinoline compared to paclitaxel. Int J Mol Sci.

[29] De Vita A, Vanni S, Fausti V, Cocchi C, Recine F, Miserocchi G (2021). Deciphering the genomic landscape and pharmacological profile of uncommon entities of adult rhabdomyosarcomas. Int J Mol Sci.

[30] Silva F, Coelho F, Peixoto A, Pinto P, Martins C, Frombach AS (2022). Establishment and characterization of a novel ovarian high-grade serous carcinoma cell line—IPO43. Cancer Cell Int.

[31] Cho EJ, Kim M, Jo D, Kim J, Oh JH, Chung HC (2021). Immuno-genomic classification of colorectal cancer organoids reveals cancer cells with intrinsic immunogenic properties associated with patient survival. J Exp Clin Cancer Res.

[32] Ries A, Flehberger D, Slany A, Pirker C, Mader JC, Mohr T (2023). Mesothelioma-associated fibroblasts enhance proliferation and migration of pleural mesothelioma cells via c-Met/PI3K and WNT signaling but do not protect against cisplatin. J Exp Clin Cancer Res.

[33] Codrich M, Dalla E, Mio C, Antoniali G, Malfatti MC, Marzinotto S (2021). Integrated multi-omics analyses on patient-derived CRC organoids highlight altered molecular pathways in colorectal cancer progression involving PTEN. J Exp Clin Cancer Res.

[34] Maier CF, Zhu L, Nanduri LK, Kühn D, Kochall S, Thepkaysone ML (2021). Patient-derived organoids of cholangiocarcinoma. Int J Mol Sci.

[35] Luo X, Fong ELS, Zhu C, Lin QXX, Xiong M, Li A (2021). Hydrogel-based colorectal cancer organoid co-culture models. Acta Biomater.

[36] Gao D, Vela I, Sboner A, Iaquinta PJ, Karthaus WR, Gopalan A (2014). Organoid cultures derived from patients with advanced prostate cancer. Cell.

[37] Kawasaki K, Toshimitsu K, Matano M, Fujita M, Fujii M, Togasaki K (2020). An organoid biobank of neuroendocrine neoplasms enables genotype-phenotype mapping. Cell.

[38] Calandrini C, Schutgens F, Oka R, Margaritis T, Candelli T, Mathijsen L (2020). An organoid biobank for childhood kidney cancers that captures disease and tissue heterogeneity. Nat Commun.

[39] Tiriac H, Belleau P, Engle DD, Plenker D, Deschênes A, Somerville TDD (2018). Organoid profiling identifies common responders to chemotherapy in pancreatic cancer. Cancer Discov.

[40] Mitra M, Mohanty C, Harilal A, Maheswari UK, Sahoo SK, Krishnakumar S (2012). A novel in vitro three-dimensional retinoblastoma model for evaluating chemotherapeutic drugs. Mol Vis.

[41] Michels BE, Mosa MH, Streibl BI, Zhan T, Menche C, Abou-El-Ardat K (2020). Pooled in vitro and in vivo CRISPR-cas9 screening identifies tumor suppressors in human colon organoids. Cell Stem Cell.

[42] Pandya PH, Jannu AJ, Bijangi-Vishehsaraei K, Dobrota E, Bailey BJ, Barghi F (2023). Integrative multi-OMICs identifies therapeutic response biomarkers and confirms fidelity of clinically annotated, serially passaged patient-derived xenografts established from primary and metastatic pediatric and AYA solid tumors. Cancers (Basel)..

[43] Wang HM, Zhang CY, Peng KC, Chen ZX, Su JW, Li YF (2023). Using patient-derived organoids to predict locally advanced or metastatic lung cancer tumor response: a real-world study. Cell Rep Med.

[44] Parmar K, Kochupurakkal BS, Lazaro JB, Wang ZC, Palakurthi S, Kirschmeier PT (2019). The CHK1 inhibitor prexasertib exhibits monotherapy activity in high-grade serous ovarian cancer models and sensitizes to PARP inhibition. Clin Cancer Res.

[45] Farago AF, Yeap BY, Stanzione M, Hung YP, Heist RS, Marcoux JP (2019). Combination olaparib and temozolomide in relapsed small-cell lung cancer. Cancer Discov.

[46] Morice PM, Coquan E, Weiswald LB, Lambert B, Vaur D, Poulain L (2021). Identifying patients eligible for PARP inhibitor treatment: from NGS-based tests to 3D functional assays. Br J Cancer.

[47] Wang Z, Gerstein M, Snyder M. RNA-Seq: a revolutionary tool for transcriptomics. Nature Reviews Genetics 2008 10:1. 2009;10(1):57–63. 10.1038/nrg2484 PMC2949280 19015660

[48] Yoon H, Lee S (2021). Integration of genomic profiling and organoid development in precision oncology. Int J Mol Sci.

[49] Tung KL, Chen KY, Negrete M, Chen T, Safi A, Aljamal AA (2021). Integrated chromatin and transcriptomic profiling of patient-derived colon cancer organoids identifies personalized drug targets to overcome oxaliplatin resistance. Genes Dis.

[50] Boretto M, Maenhoudt N, Luo X, Hennes A, Boeckx B, Bui B (2019). Patient-derived organoids from endometrial disease capture clinical heterogeneity and are amenable to drug screening. Nat Cell Biol.

[51] Chen CC, Li HW, Wang YL, Lee CC, Shen YC, Hsieh CY (2022). Patient-derived tumor organoids as a platform of precision treatment for malignant brain tumors. Sci Rep.

[52] Mo S, Tang P, Luo W, Zhang L, Li Y, Hu X (2022). Patient-derived organoids from colorectal cancer with paired liver metastasis reveal tumor heterogeneity and predict response to chemotherapy. Adv Sci.

[53] Hao M, Cao Z, Wang Z, Xin J, Kong B, Xu J (2022). Patient-derived organoid model in the prediction of chemotherapeutic drug response in colorectal cancer. ACS Biomater Sci Eng.

[54] Wu Y, Li K, Li Y, Sun T, Liu C, Dong C (2022). Grouped-seq for integrated phenotypic and transcriptomic screening of patient-derived tumor organoids. Nucleic Acids Res.

[55] Girda E, Huang EC, Leiserowitz GS, Smith LH (2017). The use of endometrial cancer patient-derived organoid culture for drug sensitivity testing is feasible. Int J Gynecol Cancer.

[56] Thrane K, Eriksson H, Maaskola J, Hansson J, Lundeberg J (2018). Spatially resolved transcriptomics enables dissection of genetic heterogeneity in stage III cutaneous malignant melanoma. Cancer Res.

[57] Shen Z, Du W, Perkins C, Fechter L, Natu V, Maecker H (2021). Platelet transcriptome identifies progressive markers and potential therapeutic targets in chronic myeloproliferative neoplasms. Cell Rep Med.

[58] Mastri M, Ramakrishnan S, Shah SD, Karasik E, Gillard BM, Moser MT (2021). Patient derived models of bladder cancer enrich the signal of the tumor cell transcriptome facilitating the analysis of the tumor cell compartment. Am J Clin Exp Urol.

[59] Bruun J, Kryeziu K, Eide PW, Moosavi SH, Eilertsen IA, Langerud J (2020). Patient-derived organoids from multiple colorectal cancer liver metastases reveal moderate intra-patient pharmacotranscriptomic heterogeneity. Clin Cancer Res.

[60] Ebisudani T, Hamamoto J, Togasaki K, Mitsuishi A, Sugihara K, Shinozaki T (2023). Genotype-phenotype mapping of a patient-derived lung cancer organoid biobank identifies NKX2–1-defined Wnt dependency in lung adenocarcinoma. Cell Rep.

[61] Kim KT, Lee HW, Lee HO, Song HJ, Jeong DE, Shin S (2016). Application of single-cell RNA sequencing in optimizing a combinatorial therapeutic strategy in metastatic renal cell carcinoma. Genome Biol.

[62] Klein AM, Mazutis L, Akartuna I, Tallapragada N, Veres A, Li V (2015). Droplet barcoding for single-cell transcriptomics applied to embryonic stem cells. Cell.

[63] Macosko EZ, Basu A, Satija R, Nemesh J, Shekhar K, Goldman M (2015). Highly parallel genome-wide expression profiling of individual cells using nanoliter droplets. Cell.

[64] Jeong YJ, Knutsdottir H, Shojaeian F, Lerner MG, Wissler MF, Henriet E (2023). Morphology-guided transcriptomic analysis of human pancreatic cancer organoids reveals microenvironmental signals that enhance invasion. J Clin Invest.

[65] Wang C, Sun M, Shao C, Schlicker L, Zhuo Y, Harim Y, et al. A multidimensional atlas of human glioblastoma organoids reveals highly coordinated molecular networks and effective drugs. bioRxiv. 2023; 2023.01.24.525374. 10.1038/s41698-024-00500-5 PMC10811239 38273014

[66] Shimura T, Toden S, Kandimalla R, Toiyama Y, Okugawa Y, Kanda M (2021). Genomewide expression profiling identifies a novel miRNA-based signature for the detection of peritoneal metastasis in patients with gastric cancer. Ann Surg.

[67] Schultz NA, Dehlendorff C, Jensen BV, Bjerregaard JK, Nielsen KR, Bojesen SE (2014). MicroRNA biomarkers in whole blood for detection of pancreatic cancer. JAMA.

[68] Chen X, Ba Y, Ma L, Cai X, Yin Y, Wang K (2008). Characterization of microRNAs in serum: a novel class of biomarkers for diagnosis of cancer and other diseases. Cell Res.

[69] Cortez MA, Calin GA (2009). MicroRNA identification in plasma and serum: a new tool to diagnose and monitor diseases. Expert Opin Biol Ther.

[70] Condrat CE, Thompson DC, Barbu MG, Bugnar OL, Boboc A, Cretoiu D (2020). miRNAs as biomarkers in disease: latest findings regarding their role in diagnosis and prognosis. Cells.

[71] Berindan-Neagoe I, Calin GA (2014). Molecular pathways: microRNAs, cancer cells, and microenvironment. Clin Cancer Res.

[72] Larson NB, McDonnell SK, Fogarty Z, Liu Y, French AJ, Tillmans LS (2022). A microRNA transcriptome-wide association study of prostate cancer risk. Front Genet.

[73] Zhang HG, Grizzle WE (2014). Exosomes: a novel pathway of local and distant intercellular communication that facilitates the growth and metastasis of neoplastic lesions. Am J Pathol.

[74] Mjelle R, Dima SO, Bacalbasa N, Chawla K, Sorop A, Cucu D (2019). Comprehensive transcriptomic analyses of tissue, serum, and serum exosomes from hepatocellular carcinoma patients. BMC Cancer.

[75] Nagai H, Kuroha M, Handa T, Karasawa H, Ohnuma S, Naito T (2021). Comprehensive analysis of microRNA profiles in organoids derived from human colorectal adenoma and cancer. Digestion.

[76] Tu J, Luo X, Liu H, Zhang J, He M. Cancer spheroids derived exosomes reveal more molecular features relevant to progressed cancer. Biochem Biophys Rep. 2021;26. 10.1016/j.bbrep.2021.101026 PMC8167213 34095553

[77] Yu LR, Stewart NA, Veenstra TD (2010). Proteomics: the deciphering of the functional genome. Essentials Gen Personalized Med.

[78] Wang K, Huang C, Nice EC. Proteomics, genomics and transcriptomics: their emerging roles in the discovery and validation of colorectal cancer biomarkers. Expert Rev Proteomics. 2014;11(2):179–205. 10.1586/14789450.2014.894466 24611605

[79] Kwon YW, Jo HS, Bae S, Seo Y, Song P, Song M (2021). Application of proteomics in cancer: recent trends and approaches for biomarkers discovery. Front Med (Lausanne).

[80] Edmondson R, Broglie JJ, Adcock AF, Yang L (2014). Three-dimensional cell culture systems and their applications in drug discovery and cell-based biosensors. Assay Drug Dev Technol.

[81] Buenafe AC, Dorrell C, Reddy AP, Klimek J, Marks DL (2022). Proteomic analysis distinguishes extracellular vesicles produced by cancerous versus healthy pancreatic organoids. Sci Rep.

[82] Frappart PO, Walter K, Gout J, Beutel AK, Morawe M, Arnold F (2020). Pancreatic cancer-derived organoids – a disease modeling tool to predict drug response. UEG J.

[83] Dijkstra JJ, Neikes HK, Rezaeifard S, Ma X, Voest EE, Tauriello DVF (2023). Multiomics of colorectal cancer organoids reveals putative mediators of cancer progression resulting from SMAD4 inactivation. J Proteome Res.

[84] Papaccio F, García-Mico B, Gimeno-Valiente F, Cabeza-Segura M, Gambardella V, Gutiérrez-Bravo MF (2023). Proteotranscriptomic analysis of advanced colorectal cancer patient derived organoids for drug sensitivity prediction. J Exp Clin Cancer Res.

[85] He W, He W, Kuang Y, Xing X, Simpson RJ, Huang H (2014). Proteomic comparison of 3D and 2D glioma models reveals increased HLA-E expression in 3D models is associated with resistance to NK cell-mediated cytotoxicity. J Proteome Res.

[86] Tölle RC, Gaggioli C, Dengjel J (2018). Three-dimensional cell culture conditions affect the proteome of cancer-associated fibroblasts. J Proteome Res.

[87] Lee SY, Park SB, Kim YE, Yoo HM, Hong J, Choi KJ, et al. iTRAQ-Based Quantitative Proteomic Comparison of 2D and 3D Adipocyte Cell Models Co-cultured with Macrophages Using Online 2D-nanoLC-ESI-MS/MS. Sci Rep. 2019; 9(1). 10.1038/s41598-019-53196-0 PMC6856061 31727937

[88] Hale LJ, Howden SE, Phipson B, Lonsdale A, Er PX, Ghobrial I (2018). 3D organoid-derived human glomeruli for personalised podocyte disease modelling and drug screening. Nat Commun.

[89] Levin VA, Panchabhai S, Shen L, Baggerly KA. Protein and phosphoprotein levels in glioma and adenocarcinoma cell lines grown in normoxia and hypoxia in monolayer and three-dimensional cultures. Proteome Sci. 2012 Jan 25;10(1). Cited 2023 Oct 17. 10.1186/1477-5956-10-5 PMC3317865 22276931

[90] Yue X, Lukowski JK, Weaver EM, Skube SB, Hummon AB (2016). Quantitative proteomic and phosphoproteomic comparison of 2D and 3D colon cancer cell culture models. J Proteome Res.

[91] Ortega-Prieto AM, Skelton JK, Wai SN, Large E, Lussignol M, Vizcay-Barrena G (2018). 3D microfluidic liver cultures as a physiological preclinical tool for hepatitis B virus infection. Nat Commun.

[92] Kuen J, Darowski D, Kluge T, Majety M (2017). Pancreatic cancer cell/fibroblast co-culture induces M2 like macrophages that influence therapeutic response in a 3D model. PLoS ONE.

[93] Aref AR, Campisi M, Ivanova E, Portell A, Larios D, Piel BP (2018). 3D microfluidic ex vivo culture of organotypic tumor spheroids to model immune checkpoint blockade. Lab Chip.

[94] Lundberg E, Borner GHH. Spatial proteomics: a powerful discovery tool for cell biology. Nature Reviews Molecular Cell Biology 2018 20:5. 2019;20(5):285–302. 10.1038/s41580-018-0094-y 30659282

[95] Marx V (2019). A dream of single-cell proteomics. Nat Methods.

[96] Hou Y, Ai X, Zhao L, Gao Z, Wang Y, Lu Y (2020). An integrated biomimetic array chip for high-throughput co-culture of liver and tumor microtissues for advanced anticancer bioactivity screening. Lab Chip.

[97] Lu X, Lodi A, Konopleva M, Tiziani S (2019). Three-dimensional leukemia co-culture system for in vitro high-content metabolomics screening. SLAS Discov.

[98] Xie P, Liang X, Song Y, Cai Z (2020). Mass spectrometry imaging combined with metabolomics revealing the proliferative effect of environmental pollutants on multicellular tumor spheroids. Anal Chem.

[99] Rodenhizer D, Gaude E, Cojocari D, Mahadevan R, Frezza C, Wouters BG (2015). A three-dimensional engineered tumour for spatial snapshot analysis of cell metabolism and phenotype in hypoxic gradients. Nat Materials.

[100] Ramachandran GK, Yeow CH (2017). Proton NMR characterization of intact primary and metastatic melanoma cells in 2D & 3D cultures. Biol Res.

[101] Palma A, Grande S, Luciani AM, Mlynárik V, Guidoni L, Viti V, et al. Metabolic Study of Breast MCF-7 Tumor Spheroids after Gamma Irradiation by (1)H NMR Spectroscopy and Microimaging. Front Oncol. 2016;6(APR). 10.3389/fonc.2016.00105 PMC4848320 27200293

[102] Liu X, Flinders C, Mumenthaler SM, Hummon AB (2018). MALDI mass spectrometry imaging for evaluation of therapeutics in colorectal tumor organoids. J Am Soc Mass Spectrom.

[103] Palubeckaitė I, Crooks L, Smith DP, Cole LM, Bram H, Le Maitre C (2020). Mass spectrometry imaging of endogenous metabolites in response to doxorubicin in a novel 3D osteosarcoma cell culture model. J Mass Spectrom.

[104] Rosi A, Grande S, Luciani AM, Barone P, Mlynarik V, Viti V (2004). (1H) MRS studies of signals from mobile lipids and from lipid metabolites: comparison of the behavior in cultured tumor cells and in spheroids. NMR Biomed.

[105] Vermeersch KA, Wang L, Mezencev R, McDonald JF, Styczynski MP (2015). OVCAR-3 spheroid-derived cells display distinct metabolic profiles. PLoS One.

[106] Coloff JL, Murphy JP, Braun CR, Harris IS, Shelton LM, Kami K (2016). Differential glutamate metabolism in proliferating and quiescent mammary epithelial cells. Cell Metab.

[107] Xiao Y, Ma D, Yang YS, Yang F, Ding JH, Gong Y (2022). Comprehensive metabolomics expands precision medicine for triple-negative breast cancer. Cell Res.

[108] Neef SK, Janssen N, Winter S, Wallisch SK, Hofmann U, Dahlke MH (2020). Metabolic drug response phenotyping in colorectal cancer organoids by LC-QTOF-MS. Metabolites.

[109] Bensaad K, Favaro E, Lewis CA, Peck B, Lord S, Collins JM (2014). Fatty acid uptake and lipid storage induced by HIF-1α contribute to cell growth and survival after hypoxia-reoxygenation. Cell Rep.

[110] Nieva C, Marro M, Santana-Codina N, Rao S, Petrov D, Sierra A (2012). The lipid phenotype of breast cancer cells characterized by Raman microspectroscopy: towards a stratification of malignancy. PLoS One.

[111] Yoshii Y, Furukawa T, Oyama N, Hasegawa Y, Kiyono Y, Nishii R (2013). Fatty acid synthase is a key target in multiple essential tumor functions of prostate cancer: uptake of radiolabeled acetate as a predictor of the targeted therapy outcome. PLoS One.

[112] Xuan Y, Wang H, Yung MMH, Chen F, Chan WS, Chan YS (2022). SCD1/FADS2 fatty acid desaturases equipoise lipid metabolic activity and redox-driven ferroptosis in ascites-derived ovarian cancer cells. Theranostics.

[113] Jones DT, Valli A, Haider S, Zhang Q, Smethurst EA, Schug ZT (2019). 3D Growth of Cancer Cells Elicits Sensitivity to Kinase Inhibitors but Not Lipid Metabolism Modifiers. Mol Cancer Ther.

[114] Xie P, Zhang J, Wu P, Wu Y, Hong Y, Wang J (2023). Multicellular tumor spheroids bridge the gap between two-dimensional cancer cells and solid tumors: the role of lipid metabolism and distribution. Chin Chem Lett.

[115] Vidavsky N, Kunitake JAMR, Diaz-Rubio ME, Chiou AE, Loh HC, Zhang S (2019). Mapping and profiling lipid distribution in a 3D model of breast cancer progression. ACS Cent Sci.

[116] Kang YP, Yoon JH, Long NP, Koo GB, Noh HJ, Oh SJ, et al. Spheroid-Induced Epithelial-Mesenchymal Transition Provokes Global Alterations of Breast Cancer Lipidome: A Multi-Layered Omics Analysis. Front Oncol. 2019;9(MAR). 10.3389/fonc.2019.00145 PMC6437068 30949448

[117] Pisanu ME, Maugeri-Saccà M, Fattore L, Bruschini S, De Vitis C, Tabbì E (2018). Inhibition of Stearoyl-CoA desaturase 1 reverts BRAF and MEK inhibition-induced selection of cancer stem cells in BRAF-mutated melanoma. J Exp Clin Cancer Res.

[118] Yang Q, Bae G, Nadiradze G, Castagna A, Berezhnoy G, Zizmare L (2022). Acidic ascites inhibits ovarian cancer cell proliferation and correlates with the metabolomic, lipidomic and inflammatory phenotype of human patients. J Transl Med.

[119] Mehrmohamadi M, Sepehri MH, Nazer N, Norouzi MR (2021). A Comparative overview of epigenomic profiling methods. Front Cell Dev Biol.

[120] Berger A, Brady NJ, Bareja R, Robinson B, Conteduca V, Augello MA (2019). N-Myc-mediated epigenetic reprogramming drives lineage plasticity in advanced prostate cancer. J Clin Invest.

[121] Lin Y, Yang Y, Yuan K, Yang S, Zhang S, Li H (2022). Multi-omics analysis based on 3D-bioprinted models innovates therapeutic target discovery of osteosarcoma. Bioact Mater.

[122] Puca L, Bareja R, Prandi D, Shaw R, Benelli M, Karthaus WR (2018). Patient derived organoids to model rare prostate cancer phenotypes. Nat Commun.

[123] Wang S, Gao S, Zeng Y, Zhu L, Mo Y, Wong CC (2022). N6-Methyladenosine reader YTHDF1 Promotes ARHGEF2 translation and RhoA signaling in colorectal cancer. Gastroenterology.

[124] Roe JS, Hwang C, Somerville TDD, Milazzo JP, Lee EJ, Da Silva B (2017). Enhancer reprogramming promotes pancreatic cancer metastasis. Cell.

[125] Sachs N, de Ligt J, Kopper O, Gogola E, Bounova G, Weeber F (2018). A living biobank of breast cancer organoids captures disease heterogeneity. Cell.

[126] Vlachogiannis G, Hedayat S, Vatsiou A, Jamin Y, Fernández-Mateos J, Khan K, et al. Patient-derived organoids model treatment response of metastatic gastrointestinal cancers. Science (1979). 2018;359(6378):920–6. 10.1126/science.aao2774 PMC6112415 29472484

[127] Roerink SF, Sasaki N, Lee-Six H, Young MD, Alexandrov LB, Behjati S (2018). Intra-tumour diversification in colorectal cancer at the single-cell level. Nature.

[128] Li X, Francies HE, Secrier M, Perner J, Miremadi A, Galeano-Dalmau N (2018). Organoid cultures recapitulate esophageal adenocarcinoma heterogeneity providing a model for clonality studies and precision therapeutics. Nat Commun.

[129] Seidlitz T, Merker SR, Rothe A, Zakrzewski F, Von Neubeck C, Grützmann K (2019). Human gastric cancer modelling using organoids. Gut.

[130] Maru Y, Tanaka N, Itami M, Hippo Y (2019). Efficient use of patient-derived organoids as a preclinical model for gynecologic tumors. Gynecol Oncol.

[131] Christensen S, Van der Roest B, Besselink N, Janssen R, Boymans S, Martens JWM, et al. 5-Fluorouracil treatment induces characteristic T>G mutations in human cancer. Nat Commun. 2019;10(1). 10.1038/s41467-019-12594-8 PMC6783534 31594944

[132] de Witte CJ, Espejo Valle-Inclan J, Hami N, Lõhmussaar K, Kopper O, Vreuls CPH, et al. Patient-Derived Ovarian Cancer Organoids Mimic Clinical Response and Exhibit Heterogeneous Inter- and Intrapatient Drug Responses. Cell Rep [Internet]. 2020 Jun 16;31(11). Cited 2023 Oct 19. 10.1016/j.celrep.2020.107762 32553164

[133] Yan HHN, Siu HC, Law S, Ho SL, Yue SSK, Tsui WY (2018). A Comprehensive human gastric cancer organoid biobank captures tumor subtype heterogeneity and enables therapeutic screening. Cell Stem Cell.

[134] Van De Wetering M, Francies HE, Francis JM, Bounova G, Iorio F, Pronk A (2015). Prospective derivation of a living organoid biobank of colorectal cancer patients. Cell.

[135] Blandin AF, Durand A, Litzler M, Tripp A, Guérin É, Ruhland E (2019). Hypoxic environment and paired hierarchical 3D and 2D models of pediatric H3.3-mutated gliomas recreate the patient tumor complexity. Cancers (Basel).

[136] Romero-Calvo I, Weber CR, Ray M, Brown M, Kirby K, Nandi RK (2019). Human organoids share structural and genetic features with primary pancreatic adenocarcinoma tumors. Mol Cancer Res.

[137] Crespo M, Vilar E, Tsai SY, Chang K, Amin S, Srinivasan T (2017). Colonic organoids derived from human induced pluripotent stem cells for modeling colorectal cancer and drug testing. Nat Med.

[138] Mullenders J, de Jongh E, Brousali A, Roosen M, Blom JPA, Begthel H (2019). Mouse and human urothelial cancer organoids: a tool for bladder cancer research. Proc Natl Acad Sci U S A.

[139] Wang J, Chen F, Liu L, Qi C, Wang B, Yan X (2016). Engineering EMT using 3D micro-scaffold to promote hepatic functions for drug hepatotoxicity evaluation. Biomaterials.

[140] Bian S, Repic M, Guo Z, Kavirayani A, Burkard T, Bagley JA (2018). Genetically engineered cerebral organoids model brain tumor formation. Nat Methods.

[141] Zhao Y, Zhang B, Ma Y, Zhao F, Chen J, Wang B (2022). Colorectal cancer patient-derived 2D and 3D models efficiently recapitulate inter- and intratumoral heterogeneity. Advanced Science.

[142] Tang-Schomer MD, Chandok H, Wu WB, Lau CC, Bookland MJ, George J (2022). 3D patient-derived tumor models to recapitulate pediatric brain tumors in vitro. Transl Oncol.

[143] Neal JT, Li X, Zhu J, Giangarra V, Grzeskowiak CL, Ju J (2018). Organoid modeling of the tumor immune microenvironment. Cell.

[144] Boj SF, Hwang C, Baker LA, Chio IIC, Engle DD, Corbo V (2015). Organoid models of human and mouse ductal pancreatic cancer. Cell.

[145] Cristobal A, van den Toorn HWP, van de Wetering M, Clevers H, Heck AJR, Mohammed S (2017). Personalized proteome profiles of healthy and tumor human colon organoids reveal both individual diversity and basic features of colorectal cancer. Cell Rep.

[146] Schumacher D, Andrieux G, Boehnke K, Keil M, Silvestri A, Silvestrov M (2019). Heterogeneous pathway activation and drug response modelled in colorectal-tumor-derived 3D cultures. PLoS Genet.

[147] Rosenbluth JM, Schackmann RCJ, Gray GK, Selfors LM, Li CMC, Boedicker M, et al. Organoid cultures from normal and cancer-prone human breast tissues preserve complex epithelial lineages. Nat Commun. 2020;11(1). 10.1038/s41467-020-15548-7 PMC7136203 32249764

[148] Benitez JA, Finlay D, Castanza A, Parisian AD, Ma J, Longobardi C (2021). PTEN deficiency leads to proteasome addiction: a novel vulnerability in glioblastoma. Neuro Oncol.

[149] Lampis A, Hahne JC, Gasparini P, Cascione L, Hedayat S, Vlachogiannis G (2021). MIR21-induced loss of junctional adhesion molecule A promotes activation of oncogenic pathways, progression and metastasis in colorectal cancer. Cell Death Differentiation.

[150] Sharick JT, Walsh CM, Sprackling CM, Pasch CA, Pham DL, Esbona K (2020). Metabolic heterogeneity in patient tumor-derived organoids by primary site and drug treatment. Front Oncol.

[151] Chen L, Dai Z, Ge C, Huang D, Zhou X, Pan K, et al. Specific metabolic response of patient-derived organoids to curcumin of colorectal cancer. J Chromatogr B Analyt Technol Biomed Life Sci. 2022;1203. 10.1016/j.jchromb.2022.123260 35598460

[152] Dornhof J, Kieninger J, Muralidharan H, Maurer J, Urban GA, Weltin A (2022). Microfluidic organ-on-chip system for multi-analyte monitoring of metabolites in 3D cell cultures. Lab Chip.

[153] Lu Y, Chen Z, Lin Q, Xia X, Lin Y, Yan J (2023). Anti-colon cancer effects of Spirulina polysaccharide and its mechanism based on 3D models. Int J Biol Macromol.

[154] Shi X, Li Y, Yuan Q, Tang S, Guo S, Zhang Y (2022). Integrated profiling of human pancreatic cancer organoids reveals chromatin accessibility features associated with drug sensitivity. Nat Commun.

[155] Wang R, Mao Y, Wang W, Zhou X, Wang W, Gao S (2022). Systematic evaluation of colorectal cancer organoid system by single-cell RNA-Seq analysis. Genome Biol.

[156] Okuno K, Pratama MY, Li J, Tokunaga M, Wang X, Kinugasa Y (2023). Ginseng mediates its anticancer activity by inhibiting the expression of DNMTs and reactivating methylation-silenced genes in colorectal cancer. Carcinogenesis.

[157] Johnson KB, Wei WQ, Weeraratne D, Frisse ME, Misulis K, Rhee K (2021). Precision medicine, ai, and the future of personalized health care. Clin Transl Sci.

[158] Malone ER, Oliva M, Sabatini PJB, Stockley TL, Siu LL (2020). Molecular profiling for precision cancer therapies. Genome Medicine.

[159] Kamps R, Brandão RD, van den Bosch BJ, Paulussen ADC, Xanthoulea S, Blok MJ (2017). Next-generation sequencing in oncology: genetic diagnosis, risk prediction and cancer classification. Int J Mol Sci.

[160] Kornauth C, Pemovska T, Vladimer GI, Bayer G, Bergmann M, Eder S (2022). Functional precision medicine provides clinical benefit in advanced aggressive hematologic cancers and identifies exceptional responders. Cancer Discov.

[161] Seppala TT, Zimmerman JW, Suri R, Zlomke H, Ivey GD, Szabolcs A (2022). Precision medicine in pancreatic cancer: patient-derived organoid pharmacotyping is a predictive biomarker of clinical treatment response. Clin Cancer Res.

[162] Hennig A, Baenke F, Klimova A, Drukewitz S, Jahnke B, Brückmann S (2022). Detecting drug resistance in pancreatic cancer organoids guides optimized chemotherapy treatment. J Pathol.

[163] Ahmad SA, Duong M, Sohal DPS, Gandhi NS, Beg MS, Wang-Gillam A (2020). Surgical outcome results from SWOG S1505: a randomized clinical trial of mFOLFIRINOX versus Gemcitabine/Nab-paclitaxel for perioperative treatment of resectable pancreatic ductal adenocarcinoma. Ann Surg.

[164] Sohal D, McDonough SL, Ahmad SA, Gandhi N, Beg MS, Wang-Gillam A (2017). SWOG S1505: A randomized phase II study of perioperative mFOLFIRINOX vs. gemcitabine/nab-paclitaxel as therapy for resectable pancreatic adenocarcinoma. J Clin Oncol.

[165] Jiang P, Sinha S, Aldape K, Hannenhalli S, Sahinalp C, Ruppin E (2022). Big data in basic and translational cancer research. Nat Rev Cancer.

[166] Creighton CJ. Making Use of Cancer Genomic Databases. Curr Protoc Mol Biol. 2018;121:19.14.1–19.14.13. 10.1002/cpmb.49 PMC5774229 29337373

[167] Chambers DA, Amir E, Saleh RR, Rodin D, Keating NL, Osterman TJ (2019). The impact of big data research on practice, policy, and cancer care. Am Soc Clin Oncol Educ Book.

[168] Jairam V, Park HS (2019). Strengths and limitations of large databases in lung cancer radiation oncology research. Transl Lung Cancer Res.

[169] Mardis ER (2021). The emergence of cancer genomics in diagnosis and precision medicine. Nat Cancer.

[170] Mittal E, Qian D. 3D organoid modeling identified that targeting IGF1R signaling may overcome drug resistance in breast cancer. bioRxiv. 2023;2023.05.14.540701.

[171] Bruna A, Rueda OM, Greenwood W, Batra AS, Callari M, Batra RN (2016). A biobank of breast cancer explants with preserved intra-tumor heterogeneity to screen anticancer compounds. Cell.

[172] Broutier L, Mastrogiovanni G, Verstegen MMA, Francies HE, Gavarró LM, Bradshaw CR (2017). Human primary liver cancer-derived organoid cultures for disease modeling and drug screening. Nat Med.

[173] Pranav P, Palaniyandi T, Baskar G, Ravi M, Rajendran BK, Sivaji A (2022). Gene expressions and their significance in organoid cultures obtained from breast cancer patient-derived biopsies. Acta Histochem.

[174] Li Y, Dou Y, Da Veiga LF, Geffen Y, Calinawan AP, Aguet F (2023). Proteogenomic data and resources for pan-cancer analysis. Cancer Cell.

[175] Wang E, Xiang K, Zhang Y, Wang XF (2022). Patient-derived organoids (PDOs) and PDO-derived xenografts (PDOXs): new opportunities in establishing faithful pre-clinical cancer models. J National Cancer Center.

[176] Nanki Y, Chiyoda T, Hirasawa A, Ookubo A, Itoh M, Ueno M (2020). Patient-derived ovarian cancer organoids capture the genomic profiles of primary tumours applicable for drug sensitivity and resistance testing. Sci Rep.

[177] Hodgson DR, Dougherty BA, Lai Z, Fielding A, Grinsted L, Spencer S (2018). Candidate biomarkers of PARP inhibitor sensitivity in ovarian cancer beyond the BRCA genes. Br J Cancer.

[178] Anderson NM, Simon MC (2020). Tumor microenvironment. Curr Biol.

[179] Colon-Echevarria CB, Lamboy-Caraballo R, Aquino-Acevedo AN, Armaiz-Pena GN (2019). Neuroendocrine regulation of tumor-associated immune cells. Front Oncol.

[180] Xu X, Farach-Carson MC, Jia X (2014). Three-dimensional in vitro tumor models for cancer research and drug evaluation. Biotechnol Adv.

