# Supplementary material for: Integration of pan-omics technologies and three-dimensional in vitro tumor models: an approach toward drug discovery and precision medicine
Source: Mol Cancer. 2024 Mar 9;23:50. doi: 10.1186/s12943-023-01916-6 (PMC10924370; doi:10.1186/s12943-023-01916-6)
Supplement: Supplementary file 1 — Additional file 1: Supplementary Table S1. Cancer databases and portals used for the analysis of pan-omics data. [file 12943_2023_1916_MOESM1_ESM.docx]

**Supplementary Table S1: Cancer databases and portals used for the analysis of pan-omics data.**

| **Sl. No.** | **Data Repositories and Analysis Portals** | **URL** | **Information Content** | **References** |
| --- | --- | --- | --- | --- |
|  | NCI Genomic Data Commons (GDC) | <https://gdc.cancer.gov/> (1) | High-quality validated datasets from the National Cancer Institute (NCI). | (2–4) |
|  | The Cancer Genome Atlas (TCGA) | <https://www.cancer.gov/ccg/research/genome-sequencing/tcga> (5) | Curated dataset for 20,000 primary cancer and comparative data with normal samples for 33 cancer types. | (6–8) |
|  | International Cancer Genomics Consortium (ICGC) | <https://dcc.icgc.org/> (9) | Mutation data catalogue of cancer. | (10,11) |
|  | TARGET (Therapeutically Applicable Research to Generate Effective Treatments) | <https://www.ncbi.nlm.nih.gov/projects/gap/cgi-bin/study.cgi?study_id=phs000218.v19.p7> (12) | Biomarkers and drug development for paediatric cancers. | (13–15) |
| 1. 5. | Clinical Proteomic Tumor Analysis Consortium (CPTAC) | <https://gdc.cancer.gov/about-gdc/contributed-genomic-data-cancer-research/clinical-proteomic-tumor-analysis-consortium-cptac> (16) | Genomic and transcript profiles. | (17,18) |
|  | Genotype-Tissue Expression (GTEx) project | <https://gtexportal.org/home/> (19) | Gene expression and regulation at tissue level. | (20,21) |
|  | Pan-cancer Analysis of the Whole Genomes (PCAWG) | <https://dcc.icgc.org/pcawg> (22) | Mutational pattern across whole cancer genomes. | (23) |
|  | Genomics Evidence Neoplasia Information Exchange (GENIE) | <https://www.aacr.org/professionals/research/aacr-project-genie/>  (24) | Publicly accessible cancer registry of real-world clinico-genomic data assembled through data sharing between 19 leading international cancer centers. | (25,26) |
|  | Human Tumor Atlas Network (HTAN) | <https://humantumoratlas.org/> (27) | Clinical, experimental, and computational data for different tumor types. | (28) |
|  | Gene Expression Omnibus (GEO) | <https://www.ncbi.nlm.nih.gov/geo/> (29) | High-throughput gene expression data. | (30) |
|  | National Cancer Data Base (NCDB) | <https://www.facs.org/quality-programs/cancer-programs/national-cancer-database/> (31) | Clinical data from hospital registries of cancer patients. | (32,33) |
|  | NCI Imaging Data Commons (IDC) | <https://portal.imaging.datacommons.cancer.gov/> (34) | Radiological imaging data for cancer patients. | (35) |
|  | The Cancer Imaging Archive (TCIA) | <https://www.cancerimagingarchive.net/> (36) | Radiological imaging data for cancer patients. | (37) |
|  | ArrayExpress | <https://www.ebi.ac.uk/biostudies/arrayexpress> (38) | High -throughput gene expression data. | (39,40) |
|  | dbGaP | <https://www.ncbi.nlm.nih.gov/gap/> (41) | Genotype and phenotype interaction data for humans. | (42,43) |
|  | Protein Atlas | <https://www.proteinatlas.org/> (44) | Protein localization and expression. | (45–47) |
|  | KEGG | [https://www.genome.jp/kegg/](about:blank) (48) | Database and analysis platform genomics and functional information. | (49,50) |
|  | cBioPortal for Cancer Genomics | <https://www.cbioportal.org/> (51) | Genomics data linked to clinical information and functional analysis. | (52–54) |
|  | COSMIC | <https://cancer.sanger.ac.uk/cosmic> (55) | Somatic mutations in cancer. | (56) |
|  | Genomics of Drug Sensitivity in Cancer (GDSC) | <http://www.cancerrxgene.org> (57) | Markers for drug sensitivity and response. | (58,59) |
|  | Framework for Data Curation (FDC) | <https://curate.ccr.cancer.gov/> (60) | Analysis of GEO and array express datasets. | (61) |
|  | Encyclopedia of DNA Elements (ENCODE) | <https://www.encodeproject.org/about/data-access/> (62) | Gene and mRNA annotations. | (63–65) |
|  | Cancer Protein Atlas (TCPA) | <https://tcpaportal.org/tcpa/> (66) | Functional proteomics data. | (67,68) |
|  | European Genome-phenome Archive (EGA) | <https://ega-archive.org/> (69) | Manually curated genetic, phenotypic, and clinical data for various diseases. | (70) |
|  | Gene Expression Database for Normal and Tumor tissues (GENT2) | <http://gent2.appex.kr/gent2/> (71) | Differential gene expression data. | (72) |
|  | canSAR | <https://cansar.ai/> (73) | Multidisciplinary database for drug discovery and research in cancer. | (74,75) |
|  | Broad GDAC Firehose | [https://gdac.broadinstitute.org/#](about:blank) (76) | Analysis portal for Cancer Genome Atlas. | (77) |
|  | canEvolve | [www.canevolve.org/](about:blank) (78) | Tumor profiles from 90 studies. | (79) |
|  | Somatic mutations altering microRNA-ceRNA interactions (SomamiR) | <https://compbio.uthsc.edu/SomamiR/> (80) | Somatic mutations in cancer. | (81,82) |
|  | UCSC Cancer Genomics Browser | <https://xenabrowser.net/> (83) | Cancer genomics and clinical data. | (84–86) |
|  | Gene Set Cancer Analysis (GSCA) | [http://bioinfo.life.hust.edu.cn/GSCA/#/](about:blank) (87) | Immunological, mutational and drug information for cancer research. | (88,89) |
|  | CTGS: Cancer Target Gene Screening | <http://ctgs.biohackers.net/> (90) | Analysis portal for multi omics datasets of breast cancer. | (91) |
|  | LOGpc (Long-term Outcome and Gene Expression Profiling Database of pan-cancers) | <https://bioinfo.henu.edu.cn/DatabaseList.jsp> (92) | Gene expression datasets. | (93) |
|  | Pan Cancer Prognostics Database (PROGgeneV2) | [http://www.progtools.net/gene/](about:blank) (94) | Ability of genes for cancer prognosis. | (95) |
|  | SurvExpress | [https://www.bitnos.com/info/survexpress](about:blank) (96) | Gene expression database and analysis portal. | (97) |
|  | PrognoScan | [http://dna00.bio.kyutech.ac.jp/PrognoScan/](about:blank) (98) | Ability of genes for cancer prognosis. | (99) |
|  | KM Plotter (Kaplan-Meier Plotter) | <https://kmplot.com/analysis/> (100) | Online tool for survival analysis. | (101) |
|  | UALCAN (University of ALabama at Birmingham CANcer data analysis Portal) | <http://ualcan.path.uab.edu/> (102) | Analysis portal for TCGA gene expression data. | (103,104) |
|  | GEPIA (Gene Expression Profiling Interactive Analysis) | <http://gepia.cancer-pku.cn/> (105) | Gene expression database and analysis portal. | (106,107) |
|  | CAS-viewer (integrative analysis of Cancer genome data based on Alternative Splicing) | [http://genomics.chpc.utah.edu/cas/](about:blank) (108) | Alternative splicing for different cancer types. | (109) |
|  | MEXPRESS | [https://mexpress.be/](about:blank) (110) | Visualization tool for TCGA data. | (111) |
|  | OncoLnc | [http://www.oncolnc.org/](about:blank) (112) | Web portal for determining interaction between TCGA data and expression levels (mRNA, miRNA and lnc RNAs). | (113) |
|  | SurvMicro | <https://bio.tools/survmicro> (114) | Analysis of miRNAs. | (115) |
|  | The Atlas of Non-coding RNA In Cancer (TANRIC) | <https://ibl.mdanderson.org/tanric/_design/basic/main.html> (116) | lncRNAs in cancer. | (117) |
|  | TIMER 2.0 | [http://timer.cistrome.org/](about:blank) (118) | Immunological infiltrates analysis in cancer. | (119) |

**References**

1. NCI Genomic Data Commons. Accessed 28 Nov 2023. https://gdc.cancer.gov/

2. Heath AP, Ferretti V, Agrawal S, An M, Angelakos JC, Arya R, et al. The NCI Genomic Data Commons. Nat Genet. 2021;53(3):257–62.

3. Zhang Z, Hernandez K, Savage J, Li S, Miller D, Agrawal S, et al. Uniform genomic data analysis in the NCI Genomic Data Commons. Nat Commun. 2021;12(1).

4. Grossman RL, Heath AP, Ferretti V, Varmus HE, Lowy DR, Kibbe WA, et al. Toward a Shared Vision for Cancer Genomic Data. N Engl J Med. 2016;375(12):1109–12.

5. The Cancer Genome Atlas Program (TCGA) - NCI. Accessed 28 Nov 2023. https://www.cancer.gov/ccg/research/genome-sequencing/tcga

6. Wang Z, Jensen MA, Zenklusen JC. A Practical Guide to The Cancer Genome Atlas (TCGA). Methods Mol Biol. 2016;1418:111–41.

7. Carrot-Zhang J, Chambwe N, Damrauer JS, Knijnenburg TA, Robertson AG, Yau C, et al. Comprehensive Analysis of Genetic Ancestry and Its Molecular Correlates in Cancer. Cancer Cell. 2020;37(5):639-654.e6.

8. Weinstein JN, Collisson EA, Mills GB, Shaw KRM, Ozenberger BA, Ellrott K, et al. The Cancer Genome Atlas Pan-Cancer analysis project. Nat Genet. 2013;45(10):1113–20.

9. ICGC Data Portal. Accessed 28 Nov 2023. https://dcc.icgc.org/

10. Zhang J, Baran J, Cros A, Guberman JM, Haider S, Hsu J, et al. International Cancer Genome Consortium Data Portal--a one-stop shop for cancer genomics data. Database (Oxford). 2011;2011.

11. Zhang J, Bajari R, Andric D, Gerthoffert F, Lepsa A, Nahal-Bose H, et al. The International Cancer Genome Consortium Data Portal. Nat Biotechnol. 2019;37(4):367–9.

12. National Cancer Institute (NCI) TARGET. Accessed 28 Nov 2023. https://www.ncbi.nlm.nih.gov/projects/gap/cgibin/study.cgi?study_id=phs000218.v19.p7

13. Mullighan CG, Su X, Zhang J, Radtke I, Phillips LAA, Miller CB, et al. Deletion of IKZF1 and prognosis in acute lymphoblastic leukemia. N Engl J Med. 2009;360(5):470–80.

14. Huang BJ, Smith JL, Farrar JE, Wang YC, Umeda M, Ries RE, et al. Integrated stem cell signature and cytomolecular risk determination in pediatric acute myeloid leukemia. Nat Commun. 2022;13(1).

15. Brady SW, Roberts KG, Gu Z, Shi L, Pounds S, Pei D, et al. The genomic landscape of pediatric acute lymphoblastic leukemia. Nat Genet. 2022;54(9):1376–89.

16. Clinical Proteomic Tumor Analysis Consortium (CPTAC) NCI Genomic Data Commons. Accessed 28 Nov 2023. https://gdc.cancer.gov/about-gdc/contributed-genomic-data-cancer-research/clinical-proteomic-tumor-analysis-consortium-cptac

17. Li Y, Dou Y, Da Veiga Leprevost F, Geffen Y, Calinawan AP, Aguet F, et al. Proteogenomic data and resources for pan-cancer analysis. Cancer Cell. 2023;41(8):1397–406.

18. Edwards NJ, Oberti M, Thangudu RR, Cai S, McGarvey PB, Jacob S, et al. The CPTAC Data Portal: A Resource for Cancer Proteomics Research. J Proteome Res. 2015;14(6):2707–13.

19. GTEx Portal. Accessed 28 Nov 2023. https://gtexportal.org/home/

20. Lonsdale J, Thomas J, Salvatore M, Phillips R, Lo E, Shad S, et al. The Genotype-Tissue Expression (GTEx) project. Nat Genet. 2013;45(6):580–5.

21. Aguet F, Barbeira AN, Bonazzola R, Brown A, Castel SE, Jo B, et al. The GTEx Consortium atlas of genetic regulatory effects across human tissues. Science. 2020;369(6509):1318–30.

22. PCAWG ICGC Data Portal. Accessed 28 Nov 2023. https://dcc.icgc.org/pcawg

23. Campbell PJ, Getz G, Korbel JO, Stuart JM, Jennings JL, Stein LD, et al. Pan-cancer analysis of whole genomes. Nature. 2020;578(7793):82–93.

24. AACR Project GENIE®: Powering Precision Medicine [Internet]. American Association for Cancer Research (AACR). [Accessed 2023 Dec 30]. Available from: https://www.aacr.org/professionals/research/aacr-project-genie

25. Sweeney SM, Cerami E, Baras A, Pugh TJ, Schultz N, Stricker T, et al. AACR Project GENIE: Powering Precision Medicine through an International Consortium. Cancer Discov. 2017;7(8):818–31.

26. Pugh TJ, Bell JL, Bruce JP, Doherty GJ, Galvin M, Green MF, et al. AACR Project GENIE: 100,000 Cases and Beyond. Cancer Discov. 2022;12(9):2044–57.

27. NCI Human Tumor Atlas Network. Accessed 28 Nov 2023. https://humantumoratlas.org/

28. Rozenblatt-Rosen O, Regev A, Oberdoerffer P, Nawy T, Hupalowska A, Rood JE, et al. The Human Tumor Atlas Network: Charting Tumor Transitions across Space and Time at Single-Cell Resolution. Cell. 2020;181(2):236–49.

29. Gene Expression Omnibus. Accessed 28 Nov 2023. https://www.ncbi.nlm.nih.gov/geo/

30. Clough E, Barrett T. The Gene Expression Omnibus Database. Methods Mol Biol. 2016;1418:93–110.

31. National Cancer Database (NCDB) ACS. Accessed 28 Nov 2023. https://www.facs.org/quality-programs/cancer-programs/national-cancer-database/

32. Bilimoria KY, Stewart AK, Winchester DP, Ko CY. The National Cancer Data Base: a powerful initiative to improve cancer care in the United States. Ann Surg Oncol. 2008;15(3):683–90.

33. Boffa DJ, Rosen JE, Mallin K, Loomis A, Gay G, Palis B, et al. Using the National Cancer Database for Outcomes Research: A Review. JAMA Oncol. 2017;3(12):1722–8.

34. IDC. Accessed 28 Nov 2023. https://portal.imaging.datacommons.cancer.gov/

35. Fedorov A, Longabaugh WJR, Pot D, Clunie DA, Pieper S, Aerts HJWL, et al. NCI Imaging Data Commons. Cancer Res. 2021;81(16):4188.

36.  The Cancer Imaging Archive (TCIA). Accessed 28 Nov 2023. https://www.cancerimagingarchive.net/

37. Clark K, Vendt B, Smith K, Freymann J, Kirby J, Koppel P, et al. The Cancer Imaging Archive (TCIA): maintaining and operating a public information repository. J Digit Imaging. 2013;26(6):1045–57.

38. ArrayExpress-Functional Genomics Data. Accessed 28 Nov 2023. https://www.ebi.ac.uk/biostudies/arrayexpress

39. Parkinson H, Kapushesky M, Shojatalab M, Abeygunawardena N, Coulson R, Farne A, et al. ArrayExpress-a public database of microarray experiments and gene expression profiles. Nucleic Acids Res. 2007;35.

40. Athar A, Füllgrabe A, George N, Iqbal H, Huerta L, Ali A, et al. ArrayExpress update - from bulk to single-cell expression data. Nucleic Acids Res. 2019;47(D1):D711–5.

41. dbGaP - NCBI. Accessed 28 Nov 2023. https://www.ncbi.nlm.nih.gov/gap/

42. Tryka KA, Hao L, Sturcke A, Jin Y, Wang ZY, Ziyabari L, et al. NCBI’s Database of Genotypes and Phenotypes: dbGaP. Nucleic Acids Res. 2014;42.

43. Wong KM, Langlais K, Tobias GS, Fletcher-Hoppe C, Krasnewich D, Leeds HS, et al. The dbGaP data browser: a new tool for browsing dbGaP controlled-access genomic data. Nucleic Acids Res. 2017;45(D1):D819–26.

44. The Human Protein Atlas. Accessed 28 Nov 2023. https://www.proteinatlas.org/

45. Thul PJ, Lindskog C. The human protein atlas: A spatial map of the human proteome. Protein Sci. 2018;27(1):233–44.

46. Uhlén M, Björling E, Agaton C, Szigyarto CAK, Amini B, Andersen E, et al. A human protein atlas for normal and cancer tissues based on antibody proteomics. Mol Cell Proteomics. 2005;4(12):1920–32.

47. Uhlén M, Fagerberg L, Hallström BM, Lindskog C, Oksvold P, Mardinoglu A, et al. Proteomics. Tissue-based map of the human proteome. Science. 2015;347(6220).

48. KEGG: Kyoto Encyclopedia of Genes and Genomes. Accessed 28 Nov 2023. https://www.genome.jp/kegg/

49. Kanehisa M, Goto S. KEGG: kyoto encyclopedia of genes and genomes. Nucleic Acids Res. 2000;28(1):27–30.

50. Jin Z, Sato Y, Kawashima M, Kanehisa M. KEGG tools for classification and analysis of viral proteins. Protein Sci. 2023;32(12):e4820.

51. cBioPortal for Cancer Genomics. Accessed 28 Nov 2023. https://www.cbioportal.org/

52. Cerami E, Gao J, Dogrusoz U, Gross BE, Sumer SO, Aksoy BA, et al. The cBio cancer genomics portal: an open platform for exploring multidimensional cancer genomics data. Cancer Discov. 2012;2(5):401–4.

53. Gao J, Aksoy BA, Dogrusoz U, Dresdner G, Gross B, Sumer SO, et al. Integrative analysis of complex cancer genomics and clinical profiles using the cBioPortal. Sci Signal. 2013;6(269).

54. de Bruijn I, Kundra R, Mastrogiacomo B, Tran TNgoc, Sikina L, Mazor T, et al. Analysis and Visualization of Longitudinal Genomic and Clinical Data from the AACR Project GENIE Biopharma Collaborative in cBioPortal. Cancer Res. 2023;83(23):3861-3867.

55. COSMIC Catalogue of Somatic Mutations in Cancer. Accessed 28 Nov 2023. https://cancer.sanger.ac.uk/cosmic

56. Tate JG, Bamford S, Jubb HC, Sondka Z, Beare DM, Bindal N, et al. COSMIC: the Catalogue Of Somatic Mutations In Cancer. Nucleic Acids Res. 2019;47(D1):D941–7.

57. Cancerrxgene - Genomics of Drug Sensitivity in Cancer. Accessed 28 Nov 2023. https://www.cancerrxgene.org/

58. Yang W, Soares J, Greninger P, Edelman EJ, Lightfoot H, Forbes S, et al. Genomics of Drug Sensitivity in Cancer (GDSC): a resource for therapeutic biomarker discovery in cancer cells. Nucleic Acids Res. 2013;41.

59. Iorio F, Knijnenburg TA, Vis DJ, Bignell GR, Menden MP, Schubert M, et al. A Landscape of Pharmacogenomic Interactions in Cancer. Cell. 2016;166(3):740–54.

60. The Framework for Data Curation (FDC). Accessed 28 Nov 2023. https://curate.ccr.cancer.gov/

61. Jiang P, Zhang Y, Ru B, Yang Y, Vu T, Paul R, et al. Systematic investigation of cytokine signaling activity at the tissue and single-cell levels. Nat Methods. 2021;18(10):1181–91.

62. ENCODE. Accessed 28 Nov 2023. https://www.encodeproject.org/about/data-access/

63. Davis CA, Hitz BC, Sloan CA, Chan ET, Davidson JM, Gabdank I, et al. The Encyclopedia of DNA elements (ENCODE): data portal update. Nucleic Acids Res. 2018;46(D1):D794–801.

64. Luo Y, Hitz BC, Gabdank I, Hilton JA, Kagda MS, Lam B, et al. New developments on the Encyclopedia of DNA Elements (ENCODE) data portal. Nucleic Acids Res. 2020;48(D1):D882–9.

65. Dunham I, Kundaje A, Aldred SF, Collins PJ, Davis CA, Doyle F, et al. An integrated encyclopedia of DNA elements in the human genome. Nature. 2012;489(7414):57–74.

66. The Cancer Proteome Atlas (TCPA). Accessed 28 Nov 2023. https://tcpaportal.org/tcpa/

67. Li J, Lu Y, Akbani R, Ju Z, Roebuck PL, Liu W, et al. TCPA: a resource for cancer functional proteomics data. Nat Methods. 2013;10(11):1046–7.

68. Chen MJM, Li J, Wang Y, Akbani R, Lu Y, Mills GB, et al. TCPA v3.0: An Integrative Platform to Explore the Pan-Cancer Analysis of Functional Proteomic Data. Mol Cell Proteomics. 2019;18(8 suppl 1):S15–25.

69. European Genome-Phenome Archive. Accessed 28 Nov 2023. https://ega-archive.org/

70. Freeberg MA, Fromont LA, D’Altri T, Romero AF, Ciges JI, Jene A, et al. The European Genome-phenome Archive in 2021. Nucleic Acids Res. 2022;50(D1):D980–7.

71. GENT2. Accessed 28 Nov 2023. http://gent2.appex.kr/gent2/

72. Park SJ, Yoon BH, Kim SK, Kim SY. GENT2: an updated gene expression database for normal and tumor tissues. BMC Med Genomics. 12 (Suppl 5), 101 (2019).

73. canSAR.ai. Accessed 28 Nov 2023. https://cansar.ai/

74. Bulusu KC, Tym JE, Coker EA, Schierz AC, Al-Lazikani B. canSAR: updated cancer research and drug discovery knowledgebase. Nucleic Acids Res. 2014;42.

75. Di Micco P, Antolin AA, Mitsopoulos C, Villasclaras-Fernandez E, Sanfelice D, Dolciami D, et al. canSAR: update to the cancer translational research and drug discovery knowledgebase. Nucleic Acids Res. 2023;51(D1):D1212–9.

76. Broad GDAC Firehose. Accessed 28 Nov 2023. https://gdac.broadinstitute.org/#

77. Deng M, Brägelmann J, Kryukov I, Saraiva-Agostinho N, Perner S. FirebrowseR: an R client to the Broad Institute’s Firehose Pipeline. Database (Oxford). 2017;2017(1).

78. canEvolve. Accessed 28 Nov 2023. https://rconnect.dfci.harvard.edu/mmreporter/

79. Samur MK, Yan Z, Wang X, Cao Q, Munshi NC, Li C, et al. canEvolve: a web portal for integrative oncogenomics. PLoS One. 2013;8(2).

80. SomamiR DB 2.0 Somatic mutations altering microRNA-ceRNA interactions. Accessed 28 Nov 2023. https://compbio.uthsc.edu/SomamiR/

81. Bhattacharya A, Ziebarth JD, Cui Y. SomamiR: a database for somatic mutations impacting microRNA function in cancer. Nucleic Acids Res. 2013;41: D977–D982.

82. Bhattacharya A, Cui Y. SomamiR 2.0: a database of cancer somatic mutations altering microRNA-ceRNA interactions. Nucleic Acids Res. 2016;44(D1):D1005–10.

83. UCSC Xena. Accessed 28 Nov 2023. https://xenabrowser.net/

84. Zhu J, Sanborn JZ, Benz S, Szeto C, Hsu F, Kuhn RM, et al. The UCSC Cancer Genomics Browser. Nat Methods. 2009;6(4):239–40.

85. Goldman MJ, Craft B, Hastie M, Repečka K, McDade F, Kamath A, et al. Visualizing and interpreting cancer genomics data via the Xena platform. Nat Biotechnol. 2020;38(6):675–8.

86. Lee CM, Barber GP, Casper J, Clawson H, Diekhans M, Gonzalez JN, et al. UCSC Genome Browser enters 20th year. Nucleic Acids Res. 2020;48(D1):D756–61.

87. GSCA: Gene Set Cancer Analysis. Accessed 28 Nov 2023. http://bioinfo.life.hust.edu.cn/GSCA/#/

88. Liu CJ, Hu FF, Xia MX, Han L, Zhang Q, Guo AY. GSCALite: a web server for gene set cancer analysis. Bioinformatics. 2018;34(21):3771–2.

89. Liu CJ, Hu FF, Xie GY, Miao YR, Li XW, Zeng Y, et al. GSCA: an integrated platform for gene set cancer analysis at genomic, pharmacogenomic and immunogenomic levels. Brief Bioinform. 2023;24(1).

90. CTGS: Cancer Target Gene Screening. Accessed 28 Nov 2023. http://ctgs.biohackers.net/

91. Kim HY, Choi HJ, Lee JY, Kong G. Cancer Target Gene Screening: a web application for breast cancer target gene screening using multi-omics data analysis. Brief Bioinform. 2020;21(2):663–75.

92. LOGpc Biomedical Informatics Institute. Accessed 28 Nov 2023. https://bioinfo.henu.edu.cn/DatabaseList.jsp

93. Wang Q, Xie L, Dang Y, Sun X, Xie T, Guo J, Han Y, Yan Z, Zhu W, Wang Y, Li W. OSlms: a web server to evaluate the prognostic value of genes in leiomyosarcoma. Frontiers in Oncology. 2019 Mar 29;9:190.

94. Pan Cancer Prognostics Database (PROGgeneV2). Accessed 28 Nov 2023. http://www.progtools.net/gene/

95. Goswami CP, Nakshatri H. PROGgeneV2: enhancements on the existing database. BMC Cancer. 2014;14(1).

96. SurvExpress. Accessed 28 Nov 2023. https://www.bitnos.com/info/survexpress

97. Aguirre-Gamboa R, Gomez-Rueda H, Martínez-Ledesma E, Martínez-Torteya A, Chacolla-Huaringa R, Rodriguez-Barrientos A, et al. SurvExpress: an online biomarker validation tool and database for cancer gene expression data using survival analysis. PLoS One. 2013;8(9).

98. PrognoScan: A new database for meta-analysis of the prognostic value of genes. Accessed 28 Nov 2023. http://dna00.bio.kyutech.ac.jp/PrognoScan/

99. Mizuno H, Kitada K, Nakai K, Sarai A. PrognoScan: a new database for meta-analysis of the prognostic value of genes. BMC Med Genomics. 2009;2:18.

100. Kaplan-Meier plotter. Accessed 28 Nov 2023. https://kmplot.com/analysis/

101. Lánczky A, Győrffy B. Web-Based Survival Analysis Tool Tailored for Medical Research (KMplot): Development and Implementation. J Med Internet Res. 2021;23(7).

102. UALCAN. Accessed 28 Nov 2023. https://ualcan.path.uab.edu/

103. Chandrashekar DS, Bashel B, Balasubramanya SAH, Creighton CJ, Ponce-Rodriguez I, Chakravarthi BVSK, et al. UALCAN: A Portal for Facilitating Tumor Subgroup Gene Expression and Survival Analyses. Neoplasia. 2017;19(8):649–58.

104. Chandrashekar DS, Karthikeyan SK, Korla PK, Patel H, Shovon AR, Athar M, et al. UALCAN: An update to the integrated cancer data analysis platform. Neoplasia. 2022;25:18–27.

105. GEPIA (Gene Expression Profiling Interactive Analysis). Accessed 28 Nov 2023. http://gepia.cancer-pku.cn/

106. Tang Z, Li C, Kang B, Gao G, Li C, Zhang Z. GEPIA: a web server for cancer and normal gene expression profiling and interactive analyses. Nucleic Acids Res. 2017;45(W1):W98–102.

107. Tang Z, Kang B, Li C, Chen T, Zhang Z. GEPIA2: an enhanced web server for large-scale expression profiling and interactive analysis. Nucleic Acids Res. 2019;47(W1):W556–60.

108. cas-viewer. Accessed 28 Nov 2023. http://genomics.chpc.utah.edu/cas/

109. Han S, Kim D, Kim Y, Choi K, Miller JE, Kim D, et al. CAS-viewer: web-based tool for splicing-guided integrative analysis of multi-omics cancer data. BMC Med Genomics. 2018;11(Suppl 2).

110. MEXPRESS. Accessed 28 Nov 2023. https://mexpress.ugent.be/

111. Koch A, Jeschke J, Van Criekinge W, Van Engeland M, De Meyer T. MEXPRESS update 2019. Nucleic Acids Res. 2019;47(W1):W561–5.

112. OncoLnc. Accessed 28 Nov 2023. http://www.oncolnc.org/

113. Anaya J. OncoLnc: Linking TCGA survival data to mRNAs, miRNAs, and lncRNAs. PeerJ Comput Sci. 2016;2016(6):e67.

114. SurvMicro bio.tools. Accessed 28 Nov 2023. https://bio.tools/survmicro

115. Aguirre-Gamboa R, Trevino V. SurvMicro: assessment of miRNA-based prognostic signatures for cancer clinical outcomes by multivariate survival analysis. Bioinformatics. 2014;30(11):1630–2.

116. TANRIC. Accessed 28 Nov 2023. https://ibl.mdanderson.org/tanric/_design/basic/main.html

117. Li J, Han L, Roebuck P, Diao L, Liu L, Yuan Y, et al. TANRIC: An Interactive Open Platform to Explore the Function of lncRNAs in Cancer. Cancer Res. 2015;75(18):3728–37.

118. TIMER2.0. Accessed 28 Nov 2023. http://timer.comp-genomics.org/timer/

119. Li T, Fu J, Zeng Z, Cohen D, Li J, Chen Q, et al. TIMER2.0 for analysis of tumor-infiltrating immune cells. Nucleic Acids Res. 2020;48(W1):W509–14.
